# Genetic Diversity, Population Structure and Differentiation of Farmed and Wild African Catfish (*Clarias gariepinus*) in Nigeria

**DOI:** 10.1111/eva.70204

**Published:** 2026-01-30

**Authors:** Mark K. Sanda, Neil B. Metcalfe, Maria Capstick, Jenna Nichols, Barbara K. Mable

**Affiliations:** ^1^ School of Biodiversity, One Health & Veterinary Medicine University of Glasgow Glasgow UK

**Keywords:** 3RAD, admixture, aquaculture, *Clarias gariepinus*, COI, fish escape

## Abstract

The African catfish ( *Clarias gariepinus* ) is a commercially important species, for both fisheries and aquaculture, and is now the most commonly farmed fish in sub‐Saharan Africa. However, knowledge about the genetic diversity and population structure of natural and farmed populations, which is crucial for effective conservation and sustainable aquaculture management, is scarce. Using mitochondrial DNA (mtDNA) cytochrome c oxidase 1 gene (COI) sequencing and genomic analysis using triple restriction site‐associated DNA sequencing (3RAD), we investigated the genetic diversity and population structure of farmed and natural *C. gariepinus* populations from Nigeria including an albino form found in the natural environment. Eleven COI haplotypes were identified, of which seven were unique to natural samples. From the 3RAD results, natural sampling sites had a slightly broader range and higher maximum values for observed heterozygosity (*H*_o_ = 0.150–0.178), expected heterozygosity (*H*_e_ = 0.173–0.213) and nucleotide diversity (*pi* = 0.181–0.228) compared to the farmed populations (*H*_o_ = 0.133–0.161, *H*_e_ = 0.116–0.149, *pi* = 0.121–0.156). Conversely, genetic differentiation (*F*_st_) was higher among farmed sampling sites compared to the natural ones and there was high genetic differentiation between the farmed and natural *C. gariepinus* sampling sites (*F*_st_ = 0.29–0.44). Admixture patterns suggested occasional mixing, possibly driven by hydrological connectivity and fish transport practices. Notably, five albino individuals sampled from the wild supported evidence of farm escapees. Outlier analyses and GO enrichment revealed loci potentially under selection related to lipid metabolism, immune signalling and apoptotic processes, indicating metabolic and immune‐related adaptations to environmental stress. Our finding of potential farm escapees highlights the potential risks associated with increasing aquaculture activities and the need for greater regulation of fish farms, which could aid monitoring and reduce the risk of escapes.

## 1 Introduction

The African catfish, also known as the African sharptooth catfish or the North African catfish, is a freshwater omnivorous species that belongs to the Claridae family and is found in tropical and subtropical climates (Omitoyin 2007). Its native distribution range spans lakes, reservoirs and rivers in sub‐Saharan Africa and it has also been introduced into South America, Southeast Asia and Europe (Konings et al. 2019; Truter et al. 2023). It is an important commercial fish both as a capture and aquaculture species, and is now the most farmed species in sub‐Saharan Africa (Chandra Segaran et al. 2023). *Clarias gariepinus* has drawn the attention of aquaculturists because of its biological attributes, which include a fast growth rate, resistance to diseases and tolerance of high stocking densities (Lal et al. 2003). Research on *C. gariepinus* has led to the development of a strain known as the ‘Dutch *Clarias*’ through selective breeding conducted in Belgium and the Netherlands, following their introduction to those countries from Africa. Dutch *Clarias* have since been introduced to different African countries, including the Central African Republic, South Africa, Côte d'Ivoire and Nigeria, where they are cultivated as farmed fish for human consumption (Huisman and Richter 1987; Holčík 1991; Roodt‐Wilding et al. 2010).

There is a tension between the use of *C. gariepinus* in aquaculture and its conservation in nature, since it is important to maintain a balance between exploiting its economic potential and preserving the genetic diversity of natural stocks. Previous studies have revealed that natural *C. gariepinus* populations exhibit high genetic variation, which is an important parameter for the species' adaptability and resilience to changing environmental conditions (Barasa et al. 2014, 2017). Given the importance of genetic diversity as a valuable resource for fish species, policy makers and managers may consider developing fisheries and aquaculture programmes that will promote the sustainable utilisation of economically important species like *C. gariepinus* . Additionally, conservation officers could benefit from addressing factors posing threats to the genetic conservation of natural resources, including loss of genetic diversity due to hybridisation with other species, as observed in Bangladesh with *C. gariepinus* and *C. batrachus* (Parvez et al. 2022), habitat fragmentation, overfishing and pollution. Additionally, the use of Dutch *Clarias* in fish farms outside their native range could negatively impact biodiversity in the event of fish escapes, as observed in Brazil and Turkey (Dumith and Santos 2022; Turan and Turan 2016).

*Clarias gariepinus* is by far the most commonly farmed fish in Nigeria (FAO 2022). Both natural‐caught *C. gariepinus* and the introduced Dutch *Clarias* are widely used in artificial breeding programmes, with reproduction being induced through hormone treatment with Ovaprim (Ataguba et al. 2009). *Clarias gariepinus* has been identified as a cheap source of animal protein and means to achieving food security, as well as job creation (Folorunso et al. 2021). Given their high economic importance to the country, it is important that the conservation of *C. gariepinus* and their sustainable exploitation in nature is prioritised. However, there is limited knowledge on the genetic diversity of either natural or farmed catfish populations in the country. This is coming at a time when fish production in Nigeria is facing threats from environmental change (evident in the receding of freshwater lakes and rivers due to extended dry seasons), overexploitation and lack of enforcement of the policy guiding fishing activities (Olopade et al. 2017). As a result, natural fish harvests in Nigeria have been declining in recent years (FAO 2022). The factors linked to the reduction in fishing yields in Nigeria have elsewhere been reported to have negative effects on the genetic diversity of natural fish populations (Kenchington 2003; Yan et al. 2021; Coleman et al. 2018). Genetic diversity is crucial for the long‐term survival, adaptation and resilience of individuals, populations, species and ecosystems, as it forms the foundation of biodiversity (Hvilsom et al. 2022). Past studies conducted on *C. gariepinus* in Nigeria have attempted to identify and assess the genetic diversity of *C. gariepinus* species using DNA markers. For example, Suleiman et al. (2020) observed high genetic diversity within farmed and natural *C. gariepinus* populations from the northeast of Nigeria using random amplified polymorphic markers (RAPD). Popoola (2022) observed high genetic differentiation among three natural *C. gariepinus* populations from southwestern Nigeria using sequences from the mitochondrial cytochrome b (cytb) gene. This molecular approach has been extended to identify natural freshwater fish in Southeastern Nigeria using the mtDNA marker cytochrome c oxidase subunit I (COI) (Nwani et al. 2011). Awodiran et al. (2019) provided useful information on genetic diversity for management and conservation of fish while attempting to differentiate between natural *C. gariepinus* populations from northcentral and southwestern Nigeria using microsatellite markers. These past studies have offered information on native species diversity but with limited geographical coverage; moreover, they did not compare farmed and natural populations to assess the levels of genetic differentiation and hybridisation, or detection of possible escapes from farmed to natural environment.

These issues can now be tackled in greater depth due to technological advances. For example, the use of high‐throughput sequencing technologies and genomic approaches in fisheries and aquaculture provide a means of identifying population structure, genetic diversity and differentiation among different fish populations, which can be used to inform fisheries management and identify escapees from fish farms (Bernatchez et al. 2017). The advancement in next‐generation sequencing has led to the assembly of over 270 fish genomes to promote studies on comparative genomics, evolution and systematics and more importantly for its application in aquaculture and fisheries (Bian et al. 2019; Hughes et al. 2018; MacKenzie and Jentoft 2016; Crollius and Weissenbach 2005; Andersson et al. 2024). However, despite its relevance as one of the most important farmed species in sub‐Saharan Africa, and decades of domestication (Hecht 2013), it was only in 2022 that the Leibniz Institute for Farm Animal Biology (FBN) sequenced and assembled the genome of *C. gariepinus* (GCA_024256425.2).

Restriction site‐associated DNA sequencing (RADseq) based on high‐throughput SNP genotyping from DNA fragmented with restriction enzyme(s) has made it possible to study a fraction of genomes (Peterson et al. 2012). The RADseq methods have evolved over the years from one restriction enzyme (Baird et al. 2008), to double digest restriction site‐associated DNA (ddRAD) sequencing (Peterson et al. 2012), through to triple‐enzyme restriction site‐associated DNA sequencing (3RAD) (Bayona‐Vásquez et al. 2019). The 3RAD approach is a low‐cost, highly robust and simple method for the construction of dual‐digest RADseq libraries using 96 pairs of Illumina compatible iTru5 and iTru7 primers enabling multiplexing of more samples and pooling of more libraries than other RADseq approaches (Bayona‐Vásquez et al. 2019). It also enables the simultaneous digestion and ligation of DNA, which minimises library construction steps. In this study, we aimed to compare the genetic diversity and differentiation among farmed and natural *C. gariepinus* populations in Nigeria, using a combination of mtDNA barcoding with the COI marker and 3RAD high‐throughput sequencing. Specifically, we assessed: (1) mtDNA diversity and differentiation between farmed and natural groups; (2) genome‐wide patterns of genetic diversity and differentiation using 3RAD and (3) genomic regions potentially under selection based on high‐resolution SNP analysis.

## 2 Methods

### 2.1 Sample Collection

**FIGURE 1:**
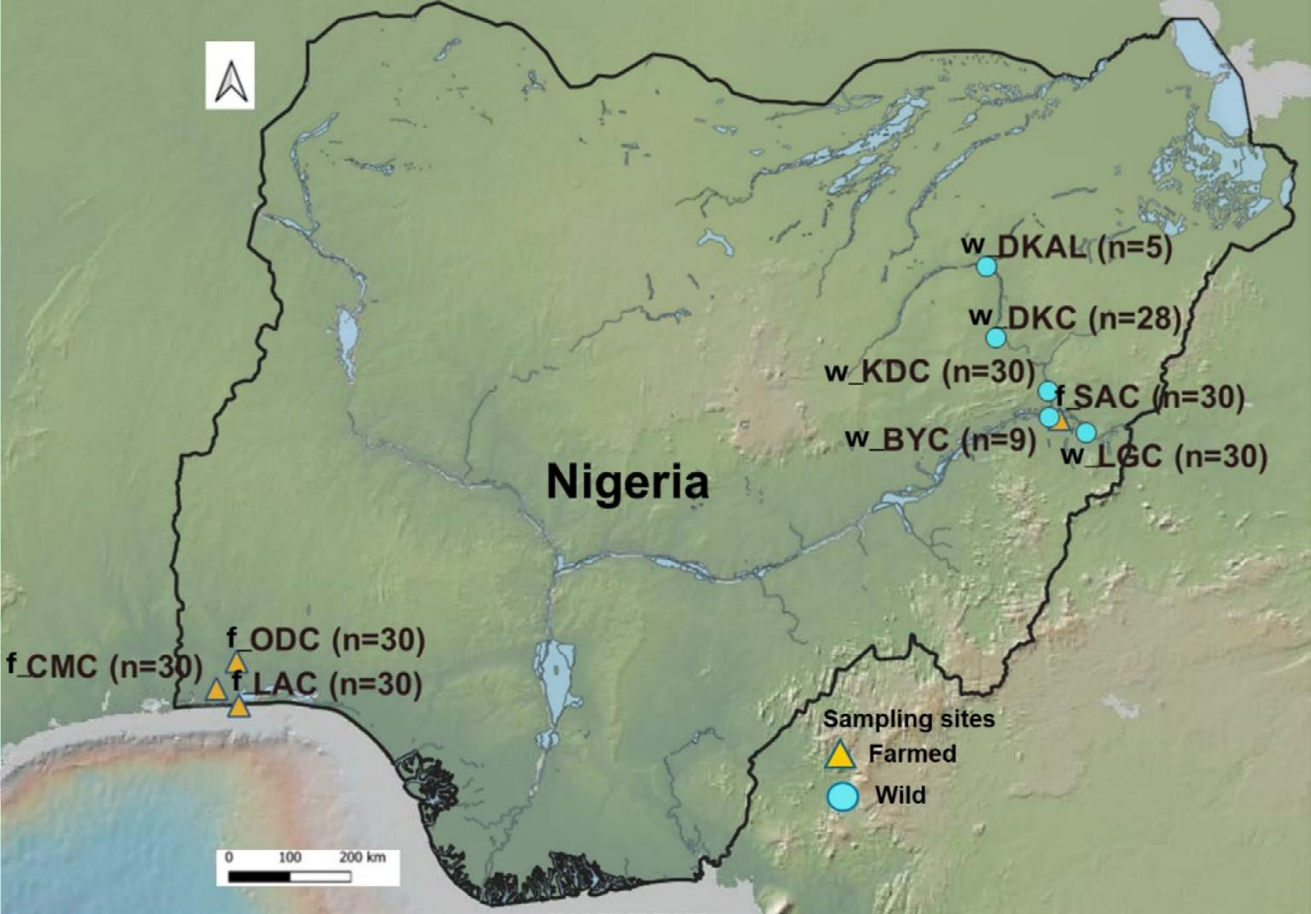
Map of Nigeria illustrating the spatial distribution of water bodies and sampling locations within the study area, categorised based on the origin of the sampled *Clarias gariepinus* populations (farmed and natural), with the sample size in parentheses. The code names are interpreted as follows: f_CMC = CMC farm, f_LAC = LAC farm, f_ODC = ODC farm, f_SAC = SAC farm, w_KDC = Kiri dam, w_LGC = Lake Geriyo, w_BYC = River Benue Yola, w_DKC = Dadin Kowa dam and w_DKAL = Albino Dadin Kowa dam.

Caudal fin clip samples of *C. gariepinus* were collected from northeastern and southwestern regions in Nigeria during a four‐month period between November 2021 and March 2022 (Figure 1, Table 1). The sampling was conducted in four farmed sites (southwest: f_CMC, f_ODC, f_LAC; northeast: f_SAC) and five natural sites in the northeast with three sites from Adamawa state (w_BYC: River Benue Yola, w_KDC: Kiri dam and w_LGC: Lake Geriyo) and two sites from Gombe state (w_DKAL: albino samples from Dadin Kowa dam, w_DKC: Dadin Kowa dam non‐albino samples). Lake Geriyo is a tributary to River Benue while Dadin Kowa dam is located on the Gongola River, which is a major tributary of the Benue River (Hassan et al. 2015; Essien et al. 2019). A total of 222 fin clips were preserved in RNAlater (Invitrogen, United Kingdom) and stored under refrigeration before transport to the UK for DNA extraction. Samples for genetic analysis were chosen by aiming for a subset of 15 randomly selected fish from each sampling site. However, the actual sample sizes varied among sites due to DNA degradation issues. For sites with sample sizes less than 15, such as the albino (*n* = 5) and River Benue Yola (*n* = 9) sampling sites, we took genetic samples from all available fish to maximise the representation of genetic diversity within these sites. This approach allowed us to adapt to the observed sample sizes and DNA quality constraints while ensuring adequate representation for downstream genetic analysis.

**TABLE 1:** Origin of farmed and natural *Clarias gariepinus* collected from various states in Nigeria, indicating the sampling locations, region, state, sampling site code and geographical coordinates, whether the population is farmed or natural, the total sample size collected per population (*N*), and sample sizes for mitochondria DNA barcoding (#COI) and 3RAD (#3RAD) analyses.

Sampling location	Region	State	Code	Latitude	Longitude	Source	*N*	#COI	#3RAD
CMC farm	Southwest	Ogun	f_CMC	6.583	3.158	Farmed	30	11	11
LAC farm	Southwest	Lagos	f_LAC	6.398	3.401	Farmed	30	11	12
Dutch *Clarias*	Southwest	Ogun	f_ODC	6.877	3.368	Farmed	30	14	12
SAC farm	Northeast	Adamawa	f_SAC	9.456	12.153	Farmed	30	12	12
Kiri dam	Northeast	Adamawa	w_KDC	9.681	12.001	Natural	30	14	10
Lake Geriyo	Northeast	Adamawa	w_LGC	9.293	12.434	Natural	30	15	12
River Benue Yola	Northeast	Adamawa	w_BYC	9.681	12.009	Natural	9	9	8
Dadin Kowa dam	Northeast	Gombe	w_DKC	10.319	11.477	Natural	28	15	13
Dadin Kowa dam^a^	Northeast	Gombe	w_DKAL	10.319	11.477	Natural	5	5	5

*Note:* The Dadin Kowa dam with an asterisk is comprised of albino samples, which were considered separately from the other samples in the population.

^a^ Albino sample.

### 2.2 DNA Isolation and PCR Amplification

Genomic DNA was extracted using DNeasy Blood & Tissue Kits (Qiagen Inc., Paisley, UK), following the manufacturer's instructions for tissue samples, with elution into 100 μL of AE buffer. The integrity of the extracted DNA was verified in 2% agarose gel electrophoresis and the concentrations (ng/μL) were measured using a Qubit 2.0 Fluorometer (Thermo Fisher Scientific, Waltham, Massachusetts, USA), with the broad‐range kit (Invitrogen, Massachusetts, USA). The cytochrome oxidase I (COI) gene was amplified using the primer pair FISH‐BCL 5′‐TCAACYAATCAYAAAGATATYGGCAC‐3′ and FISH‐BCH 5′‐TAAACTTCAGGGTGACCAAAAAATCA‐3′ (Baldwin et al. 2009). A 20 μL PCR master mix was prepared containing 15 μL ddH_2_O, 2 μL 10× buffer, 1 μL 50 mM MgCl_2_, 0.4 μL 10 mM dNTPs, 0.2 μL 10 mM primer F, 0.2 μL 10 mM primer R, 0.2 μL of 5000 units/mL Taq (Invitrogen, United Kingdom) and 1 μL DNA. PCR was performed under the following conditions: initial denaturing at 94°C for 4 min, 52°C for 50 s, 72°C for 1 min; followed by 35 cycles of 94°C for 30 s, 52°C for 30 s, 72°C for 1 min; and final extension at 72°C for 6 min and held at 10°C.

### 2.3 Mitochondrial COI Sequencing and Analysis

PCR products were sequenced using both forward and reverse primers, on an ABI 3730 automated sequencer at the University of Dundee Sequencing Service. Sequences were edited in Sequencher 5.4.6 (Gene Codes Corporation, Ann Arbor, MI, USA), aligned using the Muscle algorithm in Aliview v. 1.28 (Larsson 2014) and grouped into unique haplotypes with DnaSP v. 6 (Rozas et al. 2017). Species identification was carried out using the BOLD SYSTEMS (https://boldsystems.org/index.php) and the Basic Local Alignment Search Tool (BLAST) tool provided by the National Center for Biotechnology Information (https://blast.ncbi.nlm.nih.gov/Blast.cgi).

Haplotype frequencies were calculated and used to generate minimum spanning haplotype networks in Popart version 1.7 (Bandelt et al. 1999), both to visualise comparisons between farmed and natural sampling sites in this study and to set the Nigerian samples into a global context by comparing the geographic distributions of haplotypes with 100% match with sequences in BOLD. Arlequin v. 3.5.2.2 (Excoffier and Lischer 2010) was used to calculate summary statistics per sampling site (number of haplotypes, *N*_a_; number of segregating sites, *S*; haplotype diversity, *H*_d_; pairwise nucleotide diversity, *pi*), along with pairwise patterns of maternal genetic differentiation (*F*_st_) and hierarchical Analysis of Molecular Variance (AMOVA). To determine whether the albinos, although found in the natural environment, were more related to the farmed or natural sampling sites, results were compared across three AMOVAs: (1) with the albinos considered as natural samples; (2) without inclusion of albino samples and (3) with albinos considered as farmed samples. The AMOVAS compared variation among groups (farmed vs. natural), among populations within groups (among different sampling sites within either farmed or natural), and within populations (among individuals within each sampling site). We also used independent *t* tests to compare the mean difference between farmed and natural *C. gariepinus* when albinos were considered as natural samples, excluded from the analysis, and as farmed samples.

### 2.4 3RAD Library Preparation

Prior to library preparation, the DNA of each sample was standardised to a concentration of 20 ng/μL. The 3RAD library was prepared using 96 samples, including a negative control, using the published 3RAD protocol (Bayona‐Vásquez et al. 2019). The DNA was digested for 1 h at 37°C in a reaction mix that consisted of: 1.5 μL 10× CutSmart Buffer (New England Biolabs Inc., UK), 5.0 μL ddH_2_O, 0.5 μL of *MspI* at 20 U/μL (New England Biolabs Inc., UK), 0.5 μL of *BamHI‐HF* at 20 U/μL (New England Biolabs Inc., UK), 0.5 μL of ClaI at 20 U/μL (New England Biolabs Inc., UK), 1 μL 5 μM double‐stranded iTru read 1 adapter, 1 μL 5 μM double‐stranded iTru read 2 adapter (BadDNA, The University of Georgia, USA) and 5 μL DNA. After incubation at 37°C for 1 h, 2.0 μL dH_2_O, 1.5 μL ATP (10 μM), 0.5 μL 10× Ligase Buffer and 1.0 μL T4 DNA Ligase (100 units/μL, NEB M0202L buffer diluted 1:3 in NEB B8001S enzyme dilution buffer) were added to each reaction, before running the digested/adapter‐ligated mixtures in a thermocycler with the following conditions: 22°C for 20 min, 37°C for 10 min, 22°C for 20 min, 37°C for 10 min, 80°C for 20 min, then hold at 10°C. The ligated products were pooled by transferring 10 μL from each row in a plate (12 wells) into new 1.5 mL centrifuge tubes (10 μL × 12 well = 120 μL/row). From each of the 8 tubes containing 120 μL of ligated product, 60 μL was transferred into a single 1.5 mL tube to yield 480 μL (60 μL × 8) of ligation product. The remaining 60 μL from each strip were kept in the freezer for potential future use. The 480 μL pool was split into two (240 μL each) 1.5 mL tubes before purification.

Magnetic bead clean‐up was performed for each pool separately using NEBNext Ultra II DNA Library Prep with Sample Purification Beads (New England Biolabs Inc., UK). The clean‐up was performed at a dilution of 0.9×, followed by resuspension in 30 μL of dH_2_O. The two cleaned products were pooled into one tube (total of 60 μL). To generate full‐length library constructs, cleaned ligated products were PCR amplified in a 50 μL reaction volume containing 10.0 μL Kapa HiFi Buffer (Roche, Basel, Switzerland), 1.5 μL dNTPs (10 mM), 7.5 μL ddH_2_O, 1.0 μL Kapa HiFi DNA Polymerase (1 unit/μL), 5.0 μL iTru5 primer (5 μM), 5.0 μL iTru7 primer (5 μM) and 20 μL ligated DNA fragments from the previous step (Bayona‐Vásquez et al. 2019). Since the protocol utilises 20 μL of ligated product for the PCR, we performed three reactions from the 60 μL ligation pool. In a thermocycler, the PCR master mix was amplified using the set‐up: 98°C for 1 min; 12 cycles of 98°C for 20 s, 60°C for 15 s, 72°C for 30 s; 72°C for 5 min; hold at 15°C. To verify the success of the library preparation, we ran 5 μL of the PCR product with 2 μL loading dye on a 2.0% agarose gel for 45 min at 100 V along with a 1 kb DNA Marker (Promega, Madison, Wisconsin, USA). A successful library preparation was indicated by a bright evenly distributed DNA smear around ~300–800 bp. All three PCR products were pooled purified with NEBNext Ultra II DNA Library Prep Sample Purification Beads in a 1:1.5 (DNA:Beads) ratio and cleaned DNA was eluted in 44 μL of ddH_2_0. The purified product was quantified using a Qubit Fluorimeter broad‐range assay (Thermo Fisher Scientific, Waltham, Massachusetts, USA).

#### 2.4.1 Size Selection and Sequencing

The pooled library was size‐selected using a Pippin Prep (Sage Science, Beverly, MA, USA) with a 2% dye‐free Marker L agarose gel cassette (CDF2010), set to capture fragments of 300–450 bp, and eluted in 40 μL of Tris‐TAPS (N‐[tris (hydroxymethyl) methyl]‐3‐amino propane sulfonic acid) buffer. The eluted library was quantified with the Qubit high‐sensitivity assay (Thermo Fisher Scientific, Waltham, Massachusetts, USA) and sequenced (150 bp paired‐end) on a single lane Novaseq X (Illumina, San Diego, California, USA) at Novogene Co. Ltd. (Cambridge, UK).

#### 2.4.2 3RAD Sequencing Analysis

Raw reads were demultiplexed into individual samples using the process_radtags module in Stacks V2.65 (Rivera‐Colón and Catchen 2022). Filtering parameters were set to drop reads with a Phred quality score of 20 or less and to remove any reads with uncalled bases. The quality of the demultiplexed reads including base quality scores, per‐based sequence content, per‐sequence quality scores and sequence length distribution were assessed using FastQC (Andrews 2010). Reads were mapped to the *C. gariepinus* reference genome GCF_024256425.1 using BWA‐MEM (Li and Durbin 2009). We conducted two population analyses in Stacks V2.65 (Rivera‐Colón and Catchen 2022). The first used relaxed parameters to capture the number of SNPs per locus. In this analysis, we specified that the populations program remove loci that could not be called in more than 80% of individuals per study site. After assessing the distribution of the number of SNPs per locus, loci with single SNPs were retained in a whitelist (loci to keep) and passed as an input file in the second population analysis with stricter filtering parameters, instructing the program to: (1) only include loci present in all populations (nine farmed and natural sites); (3) remove loci that could not be called in more than 80% of individuals per study site; (4) remove loci with a minor allele frequency below 0.05; (5) remove loci that deviate significantly from Hardy–Weinberg equilibrium and (6) retain a single SNP per RAD per locus (–*write‐single‐snp*), therefore excluding SNPs that are physically linked. Summary statistics (*H*_o_, *H*_e_ and *pi*) and inbreeding coefficients based on the difference between *H*_o_ and *H*_e_ (*F*_is_) were computed for each sampling site and compared between farmed and natural sites.

Using the linkage disequilibrium method in NeEstimator v2, the effective population size (*N*_e_) was estimated for all sampling sites (Do et al. 2014). The estimate was based on SNP loci filtered to include only alleles with a minor allele frequency ≥ 0.05, which helps reduce the bias caused by rare alleles.

#### 2.4.3 Genetic Differentiation, Population Structure and Admixture

Pairwise *F*_st_ was calculated between each pair of sampling sites using Genepop output from the Stacks *populations* analysis (Rivera‐Colón and Catchen 2022). To assess genetic differentiation among sampling sites, *F*_st_ values were calculated using the *hierfstat* (Goudet 2005) and adegenet (Jombart et al. 2023) packages in R (R Core Team 2018). The Genepop file was read into R and converted to a genind object to compute pairwise *F*_st_ values using the genet.dist function with the ‘WC84’ method for implementation of Weir and Cockerham (1984) *F*_st_. Clustered heatmaps based on the *F*_st_ values were generated using the Euclidean distance function from the *pheatmap* package in R (Kolde 2019). Using the *boot.ppfst* function, a permutation test with 1000 bootstrap replicates was performed to assess the significance of the reported *F*_st_ values (Goudet 2005).

To visualise genetic variation with and between individuals in the farmed and natural groups, principal components analysis (PCA) was conducted based on allele frequencies from data contained in the *Genepop* file generated from Stacks populations analysis. The *Genepop* file was loaded into R using the *Adegenet* package and converted into a *GenInd* object (Jombart 2008). The *GenInd* object was used to generate a population sample table with individual samples and population ID and converted into a dataframe. To visualise genetic variation and relatedness among individuals within and differentiation across sampling sites through patterns of clustering, we used the scale function *scaleGen* and *dudi.pca*. PCA eigenvalues were added to the dataframe, and a scatterplot function was performed using *ggplot2* package in R (Wickham 2016) to visualise principal components 1 and 2, which explained most of the genetic variation in the data. The *scale_color_manual* function in *ggplot2* package was used to assign unique colours to differentiate the different sampling sites in the cluster.

Admixture analysis was conducted using the ADMIXTURE software (Alexander et al. 2009). To infer the optimal number of ancestral populations (*K*) contributing to the genetic structure of the *C. gariepinus* sampling sites, cross‐validation was performed for *K* values ranging from 2 to 10, using 1000 bootstrap replicates and 3 iterations. The *K* value associated with the lowest cross‐validation error was selected as the best estimate of population structure. *K* = 2 was also assessed to test the hypothesis that farmed individuals can be differentiated from natural ones.

#### 2.4.4 SNP Outlier Analysis

Outlier analysis was conducted to detect genomic regions potentially under selection, using three different approaches to investigate SNPs from the second population analysis. In the first approach, the R package *PCAdapt* (Privé et al. 2020; Luu et al. 2017) was used to perform genome scans to detect genes under selection without needing to pre‐define populations. It uses PCA to infer population structure directly from the genetic data (in this case the SNP data in VCF format), and then identifies SNPs that are unusually associated with that population structure (Luu et al. 2017). The test statistic for detecting outliers was then computed based on regressing SNPs against the selected value of *K* principal components (*z* score) and comparing scaled Mahalanobis distances between the point for each SNP and the mean, with *p*‐values calculated based on a chi‐square distribution with *K* degrees of freedom under an assumption of no outliers. Adjusted *p‐*values were calculated using the Bonferroni method to control the likelihood of obtaining false positives, specifying a 5% significance level (*α* = 0.05). The −log_10_ of the *p*‐values were visualised in a Manhattan plot to detect outliers across the genome using *q*‐values < 0.05 for significant SNPs.

The second approach used VCFtools (Danecek et al. 2011) to detect outliers based on *F*_st_ calculated between pairs of populations: (1) farmed vs. natural (excluding albinos) and (2) farmed vs. natural (including albinos), and albino vs. farmed. This group comparison aimed to identify genomic regions potentially shaped by artificial selection in farmed populations relative to natural populations, without the confounding influence of suspected escapees (albinos). SNPs with *F*_st_ values that fell outside a 95% threshold range were classified as outliers and flagged as potential genomic regions under selection or candidates for adaptive evolution.

Finally, *Bayescan* was used to identify outlier SNPs with high *F*_st_ using a Bayesian approach (Foll and Gaggiotti 2008). The VCF file was converted to GESTE/BayeScan format using PGDSpider v. 3.0 (Lischer and Excoffier 2011), and independent *Bayescan* runs were executed using posterior odds of 100 to test how likely it is that a locus is under selection versus neutrality. The following default parameters were used: iterations = 5000, thinning interval size = 10, number of pilot runs = 20, length of pilot = 5000 and burn‐in length = 50,000. Alpha coefficients were estimated to show the strength and direction of selection, where a positive value suggests diversifying selection, whereas negative values suggest balancing or purifying selection and 0 indicates neutrality (Foll and Gaggiotti 2008). Alpha coefficients also indicate how much the allele frequency at a locus deviates from neutral expectations. *α* values ≥ 0 and *q*‐value ≤ 0.05 suggest significant positive (diversifying) selection; *α* values ≥ 0 and *q*‐value > 0.05 will indicate neutral selection, whereas *α* < 0 suggests balancing selection.

Over‐representation analysis was conducted to define functions of detected outliers and their relationships with one another. The null set of GO annotations used to determine enrichment included all genes in the genome that had significant SNPs represented in the dataset after quality filtering. The web server g:Profiler was used to carry out gene ontology analysis to map the list of outlier genes to known functional information sources. It also detected statistically significantly enriched biological process (BP) that describe in which biological process the gene product participates, and cellular components (CC) that describe in which part of the cell the particular gene product is physically located (Raudvere et al. 2019).

## 3 Results

### 3.1 mtDNA Haplotype Distribution and Diversity in Farmed and Natural *C. gariepinus* Populations

**FIGURE 2:**
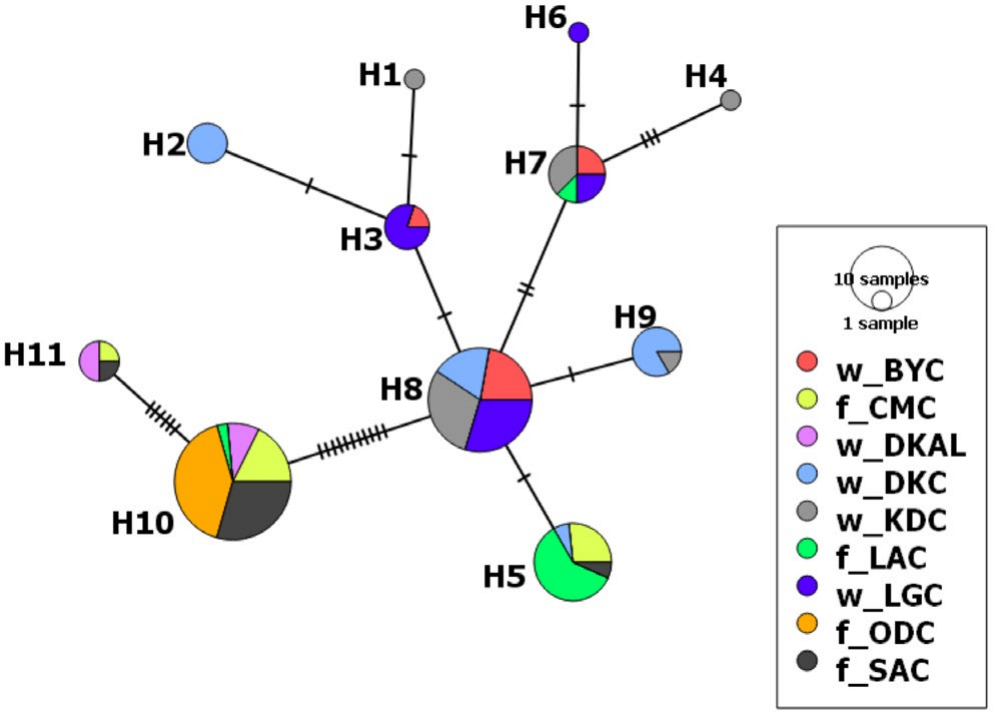
Haplotype network showing genetic relationships of farmed (f_) and natural (w_) *Clarias gariepinus* populations from Northeast (w_BYC, w_DKAL, w_DKC, w_KDC, w_LGC and f_SAC) and Southwest (f_CMC, f_LAC, f_ODC) Nigeria. Haplotype 8, shared among w_KDC, w_LGC, w_BYC and w_DKC, appears to be the ancestral haplotype, with the most connections to other haplotypes. The albino *C. gariepinus* from Dadin Kowa dam (w_DKAL; purple) is the only natural population which had the farmed‐dominated haplotypes 10 and 11.

**FIGURE 3:**
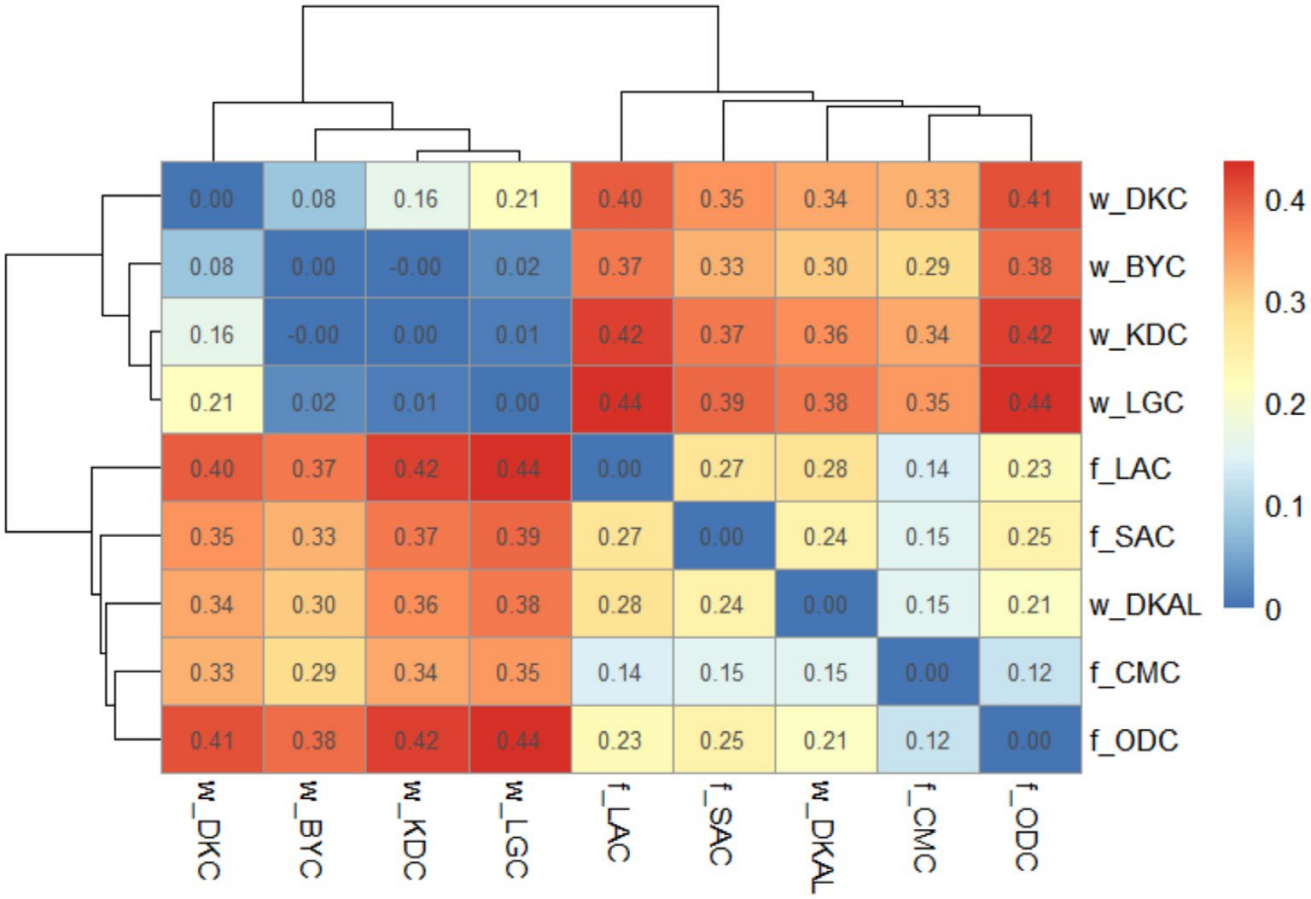
Pairwise genetic differentiation (*F*_st_) among nine *Clarias gariepinus* from farmed and natural populations based on SNP data. f_CMC: CMC farm, f_ODC: Dutch Clarias, f_LAC: Lagos farm, f_SAC: SAC farm, w_BYC: River Benue Yola, w_LGC: Lake Geriyo, w_DKAL: Albino Dadin Kowa, w_DKC: Dandin Kowa dam and w_KDC: Kiri dam. The dendrogram displays the structure of genetic similarity among sampling sites, with more closely related sampling sites clustering together on nearby branches. The heatmap shows the degree of genetic differentiation between two sampling sites increasing from very low (blue) to very high (red) genetic differentiation.

Overall, 104 COI sequences of between 500 and 680 bp identified as *C. gariepinus* (GenBank accession numbers: PQ197861–PQ197964) were obtained and collapsed to eleven unique haplotypes. Species identification was performed on the BOLD Systems and BLAST search engines, and both databases returned a match for *C. gariepinus* with similarity scores ranging from 99.68% to 100% (Table 2). The aligned sequences had a mean nucleotide composition of C = 26.19%, T = 28.67%, A = 27.45% and G = 17.69%. All haplotypes were observed in natural samples, but only four were identified in farmed fish. Two farmed haplotypes (H10, H11) were shared exclusively with albino ‘natural’ samples (w_DKAL). H10 dominated farmed samples (31 out of 43), whereas H8 was most frequent in natural sites (27/58). H8 appeared to be ancestral and had numerous connections to other natural haplotypes and the most frequently identified farmed haplotype (H10) (Figure 2, 3). Haplotypes 1–4, 6, 8 and 9 were unique to natural sampling sites.

**TABLE 2:** Haplotype identification table based on the Barcode of Life Database (BOLD) showing haplotype ID, sample sizes (*N*), species similarity percentage and source classification (farmed/natural).

Haplotype ID	*N*	Farmed	Natural	Similarity	Source
1	1	—	w_KDC (1)	99.83	Natural
2	4	—	w_DKC (4)	99.68	Natural
3	5	—	w_BYC (1), w_LGC (4)	99.84	Natural
4	1	—	w_KDC (1)	99.82	Natural
5	15	f_CMC (4), f_LAC (9)	w_KDC (1)	100	Farmed/natural
6	1	—	w_LGC (1)	99.84	Natural
7	8	f_LAC (1)	w_BYC (2), w_KDC (3), w_LGC (2)	100	Farmed/natural
8	27	—	w_BYC (6), w_DKC (5), w_KDC (8), w_LGC (8)	100	Natural
9	6	—	w_DKC (5), w_KDC (1)	100	Natural
10	34	f_CMC (6), f_LAC (1), f_ODC (14), f_SAC (10)	w_DKAL (3)	100	Farmed/natural
11	4	f_CMC (1), f_SAC (1)	w_DKAL (2)	100	Farmed/natural

The BOLD Systems search results for haplotypes that had a 100% match (haplotypes 5, 7–11) revealed that these had global distributions in samples from fish farms across Africa, Asia and the Middle East (Table S1). Haplotypes 5, 7 and 8 had previously been found in Nigeria, based on previous studies conducted in the country (Nwani et al. 2011; Iyiola et al. 2018). Haplotype 8, frequently found in our natural fish samples, was also found in farm samples from Israel, Thailand, Bangladesh, Syria and India. In contrast, the frequent farmed haplotype 10 was found in DR Congo and Brazil (Table S1).

### 3.2 mtDNA Population Genetic Diversity

Based on the COI Sanger sequences, fish from natural sampling sites (including the albino w_DKAL) had more haplotypes (ranging from 2 to 5; average = 3.6) than those from farmed sites (ranging from 1 to 3; average = 2.5), with the highest number in natural site w_KDC (*n* = 5) and only a single haplotype in samples from farm site f_ODC (Table 3). There was no significant difference in the number of haplotypes (*N*_a_) between the farmed and the natural groups if the albino w_DKAL samples were considered to be a natural population, nor if those samples were excluded (Table S2). However, when the albino population was included with the farmed sampling sites, there was a significant difference between farmed and natural sites. The farmed sampling sites exhibited a non‐significant trend for more segregating sites than natural sampling sites regardless of whether albinos were considered as natural, not included in the analysis, or considered as farmed. Haplotype diversity (*H*_d_) tended to be higher in natural populations (average = 0.646; highest *H*_d_ observed in w_DKC) compared to farmed (average = 0.321) sampling sites, except for f_CMC, which had higher *H*_d_ than some of the farmed sampling sites. However, the differences were not significant, regardless of whether albino samples were considered as natural, excluded from the analysis or considered as farmed. Pairwise nucleotide diversity (*pi*) was highest in the f_CMC population and overall tended to be higher in the farmed (average = 0.005) than the natural (average = 0.003) populations, except for the albino samples, which showed *pi* more similar to the farmed sampling sites (w_DKAL = 0.006). However, differences between farmed and natural were not significant, even when the albinos were considered as farmed.

**TABLE 3:** Mitochondrial DNA variation of farmed and natural African catfish ( *Clarias gariepinus* ) collected from various states in Nigeria, indicating the sampling site (population) and code, region, the type of population (source), number of haplotypes (*N*_a_), number of segregating sites (*S*), haplotype diversity (*H*_d_) ± standard deviation (SD) and pairwise nucleotide diversity (*p* *i* ± SD).

Population	Code	Region	Source	*N*_a_	*S*	*H*_d_ ± SD	*pi* ± SD
CMC farm	f_CMC	Southwest	Farmed	3	18	0.618 ± 0.104	0.012 ± 0.007
LAC farm	f_LAC	Southwest	Farmed	3	14	0.346 ± 0.172	0.004 ± 0.003
Dutch *Clarias*	f_ODC	Southwest	Farmed	1	—	—	—
SAC farm	f_SAC	Northeast	Farmed	3	18	0.318 ± 0.164	0.005 ± 0.003
Kiri dam	w_KDC	Northeast	Natural	5	7	0.659 ± 0.123	0.003 ± 0.002
Lake Geriyo	w_LGC	Northeast	Natural	4	4	0.667 ± 0.099	0.002 ± 0.002
River Benue Yola	w_BYC	Northeast	Natural	3	3	0.556 ± 0.163	0.002 ± 0.001
Dadin Kowa dam	w_DKC	Northeast	Natural	4	4	0.752 ± 0.056	0.002 ± 0.002
Dadin Kowa dam^a^	w_DKAL	Northeast	Natural	2	6	0.600 ± 0.175	0.006 ± 0.004

^a^ Albino sample.

### 3.3 mtDNA COI Genetic Differentiation Among Populations

In the AMOVA analyses, the comparison of groups (farmed vs. natural) did not explain a significant proportion of the variation, with the majority of the variation being found among individuals within populations, followed by among populations within the farmed and natural sampling sites. This finding was regardless of whether albinos were considered as natural samples (Table S3), excluded (Table S4), or considered as farmed (Table S5). A global AMOVA revealed significant genetic differentiation among populations (*F*_st_ = 0.408, *p* = 0.001).

### 3.4 3RAD Reads Summary

The demultiplexed library resulted in 1,583,447,229 reads, ranging from 1,198,636 to 35,931,402 per individual. All samples passed the FASTQ check, with a sequence per base quality score ≥ 28. The mean read coverage was 109.5× (Figure S1a) but with varying distribution of reads across sampling sites (Figure S1b). A total of 1,663,643 loci were assembled, ranging from 37,000 to 100,000 loci per sample (Figure S2a). The mean rate of missing variant sites (i.e., the average percentage of genomic locations that do not have any sequence information) within sampling sites was less than 0.05% for most samples, except for one sample from the w_DKC sampling site with about 25% missing variant sites (Figure S2b). This sample was dropped from subsequent analysis. The first STACKS populations analysis revealed the distribution of SNPs (Figure S3) and retained 68,344 SNPs, whereas a more conservative run of loci with only one SNP per locus retained 767 high‐confidence SNPs. This reduction reflects a deliberate attempt to ensure that only high‐resolution SNPs were retained for accurately assessing genetic diversity, population structure and differentiation.

### 3.5 Population Summary Statistics

Based on the 767 variant sites retained, natural samples (excluding the albinos) tended to show higher maximum values and broader ranges for *H*_o_ (0.150–0.178), *H*_e_ (0.173–0.217) and *pi* (0.181–0.226) compared to the farmed sites (*H*_o_ = 0.133–0.161, *H*_e_ = 0.116–0.149, *pi* = 0.121–0.156) (Table 4). There was no significant difference in average *Ho* between farmed (*H*_o_ = 0.144) and natural (*H*_o_ = 0.163) sites (*t* = −2.192, df = 5.977, *p* = 0.0710). However, average *H*_e_ and *pi* in the farmed sites (*H*_e_ = 0.131; *pi* = 0.137) were significantly different from the natural (*H*_e_ = 0.197; *pi* = 0.208) sites (*t* = −4.939, df = 5.523, *p* = 0.003; *t* = −4.926, df = 5.506, *p* = 0.003, respectively). When the albino samples were included as farmed samples in the analyses, all the summary statistics including *H*_o_ were significantly different in a mean comparison between farmed and natural sampling sites. However, when albino samples were considered as natural, the mean difference between farmed and natural sampling sites for *H*_o_, *H*_e_ and *pi* was not significantly different.

**TABLE 4:** Summary of observed (*H*_o_) and expected heterozygosity (*H*_e_), nucleotide diversity (*pi*), inbreeding coefficients (*F*_is_) and effective population size (*N*_e_) estimated from 767 variant sites, showing the genetic diversity in farmed (f_) and natural (w_) *Clarias gariepinus* in Nigeria.

Pop ID	Code	*N*	*H*_o_	*H*_e_	*p* *i*	*F*_is_	*N*_e_
CMC farm	f_CMC	10	0.161	0.149	0.156	−0.006	10.2
Lagos farm	f_LAC	12	0.142	0.120	0.125	−0.035	83.2
Ogun Dutch	f_ODC	12	0.141	0.116	0.121	−0.043	∞
SAC	f_SAC	11	0.133	0.140	0.147	0.037	2.0
River Benue	w_BYC	9	0.169	0.213	0.228	0.155	2.5
Dadin Kowa Albino	w_DKAL	5	0.110	0.105	0.118	0.023	3.4
Dadin Kowa	w_DKC	11	0.178	0.217	0.226	0.134	1.4
Kiri Dam	w_KDC	8	0.150	0.185	0.196	0.143	7.4
Lake Geriyo	w_LGC	12	0.156	0.173	0.181	0.076	9497.7

In terms of inbreeding, the farmed f_CMC, f_LAC and f_ODC samples had a slight excess of heterozygosity (i.e., negative inbreeding coefficient, *F*_is_), whereas all the other farmed samples were positive. For natural samples, there was only substantial evidence of inbreeding in the w_BYC, w_KDC and w_DKC sites (values of *F*_is_ > 0.1); the albino (w_DKAL) samples showed the lowest inbreeding coefficient (*F*_is_ = 0.023) among the natural sites. Even when albino samples were considered as natural, inbreeding coefficients were significantly lower in farmed (*F*_is_ = −0.012) compared to natural (*F*_is_ = 0.106) sites (*t* = −3.838, df = 6.813, *p* = 0.007). When albino samples were considered as farmed, the average increased for farmed (*F*_is_ = −0.005) and decreased for natural (*F*_is_ = 0.127) sites but the difference remained significant (*t* = −5.609, df = 6.558, *p* = 0.001).

The effective population size (*N*_e_) was predicted to be extremely low (less than the number of samples) in both farmed and wild populations (Table 4), except for Lagos farm (f_LAC) and Lake Geriyo (w_LGC). The *N*_e_ for the f_ODC sampling site could not be estimated using the linkage disequilibrium method, as the estimate was infinite (Table 4).

### 3.6 Genetic Differentiation and Admixture Between Farmed and Natural Populations

The pairwise *F*_st_ comparisons between sampling sites from the 3RAD genotypic data showed lower genetic differentiation among the natural sampling sites (0–0.21) compared to the farmed sites (0.12–0.27) (Figure 3). Pairwise *F*_st_ comparisons among natural sampling sites were significant except for the comparison between w_BYC and w_KDC (*F*_st_ [95% bootstrapped CI] = −0.00 [−0.010 to 0.007]). *F*_st_ was also significant for paired comparisons among the farmed sampling sites. Comparisons between the albino sample (w_DKAL) and the farmed sampling sites showed consistently low but significant *F*_st_ values, ranging from 0.15 (95% bootstrapped CI = 0.213–0.1185) when w_DKAL was compared to f_CMC to 0.28 (CI = 0.244–0.315) when compared to f_LAC. However, *F*_st_ values were consistently higher when the albino sample was compared to other natural sites, ranging from 0.30 (CI = 0.273–0.388) when comparing w_DKAL to w_BYC to 0.38 (CI = 0.342–0.412) when compared to w_LGC. The lowest *F*_st_ (−0.00) among the natural sampling sites was observed between Kiri Dam (w_KDC) and River Benue Yola (w_BYC). Hierarchical clustering analysis based on the *F*_st_ heatmap (Figure 3) indicated one large cluster consisting of the farmed sites together with the albino w_DKAL site and a second cluster containing the remainder of the natural sampling sites. The figure also indicates low genetic differentiation among natural sampling sites in Adamawa state (w_BYC, w_KDC and w_LGC), ranging from *F*_st_ = −0.00 (between w_BYC and w_KDC) to *F*_st_ = 0.02 (between w_BYC and w_LGC). This contrasts with much higher genetic differentiation when these Adamawa sampling sites were compared with the w_DKC site in Gombe state (*F*_st_ values ranging from 0.08 to 0.21).

**FIGURE 4:**
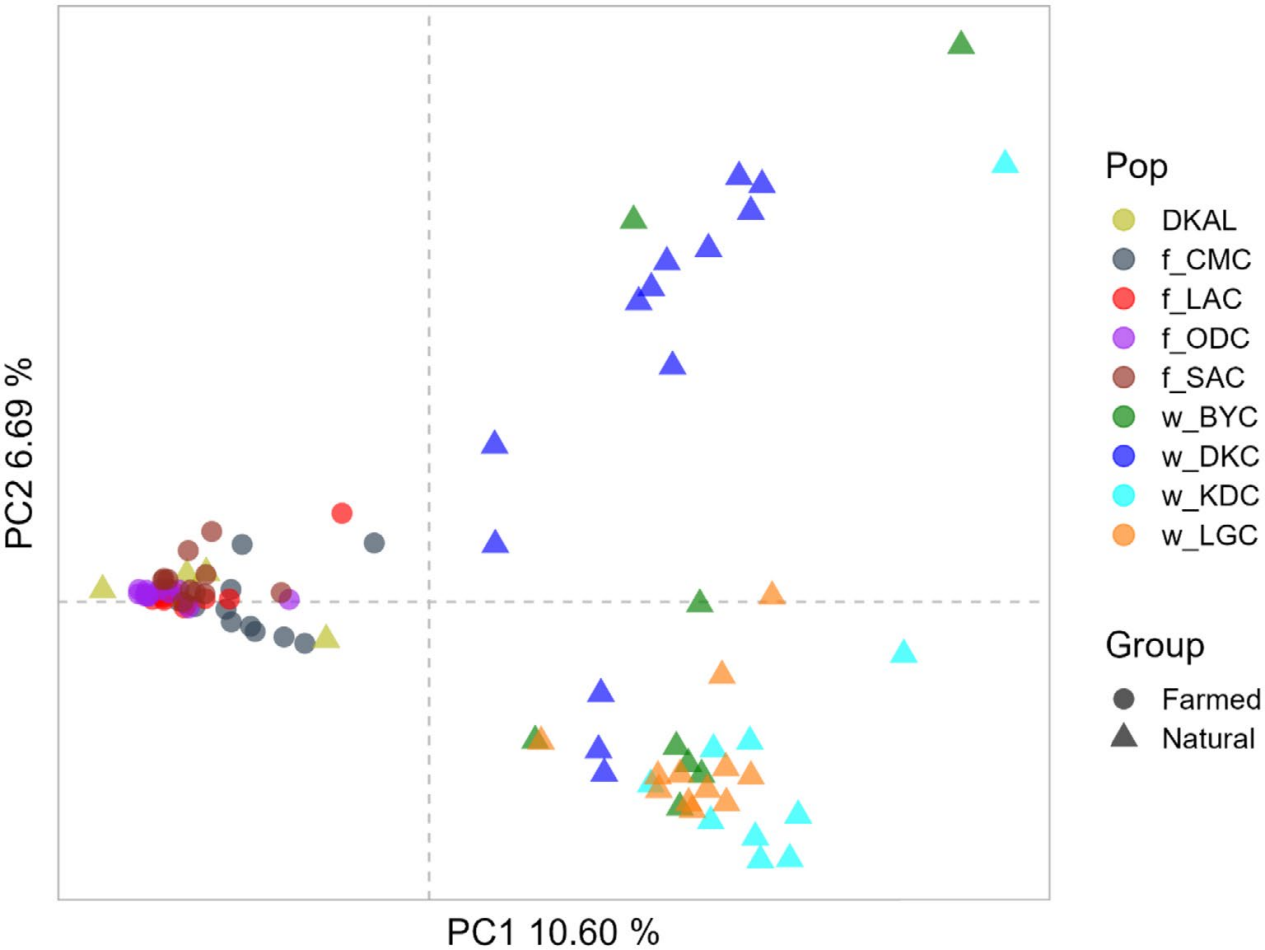
Principal component analysis (PCA) plot of farmed and natural *Clarias gariepinus* generated from SNP data. The *x*‐axis and *y*‐axis represent the first and second principal components, respectively, which are linear combinations of SNP alleles that capture the maximum amount of variation in the data. PC1 (explaining 20.72% of the variation) separates the natural samples (triangles) from the farmed samples (circles), except for the natural albino population (w_DKAL) sampled from Dadin Kowa dam. PC2 (explaining 13.26% of the variance) separates some of the natural populations from one another; note that w_BYC is found in both main natural clusters.

The PCA plot also showed a clear separation between the natural and farmed samples, with principal components 1 (PC 1) and 2 (PC 2) explaining 10.60% and 6.69% of the variance, respectively (Figure 4). The distribution of individuals and sampling sites on the PCA plot showed a major cluster of all the farmed samples along the PC1 axis, but with the albino samples again clustering with them rather than with natural samples. Among the natural sampling sites, samples from Adamawa state including all individuals from w_LGC and the majority of the w_BYC and w_KDC samples clustered at the bottom (i.e., lower values of PC2). At the top of the PC 2 axis is one major cluster consisting of the Gombe samples (w_DKC: Dadin Kowa dam), another two w_DKC samples that were loosely clustered, and one outlier each from w_BYC and w_KDC.

**FIGURE 5:**
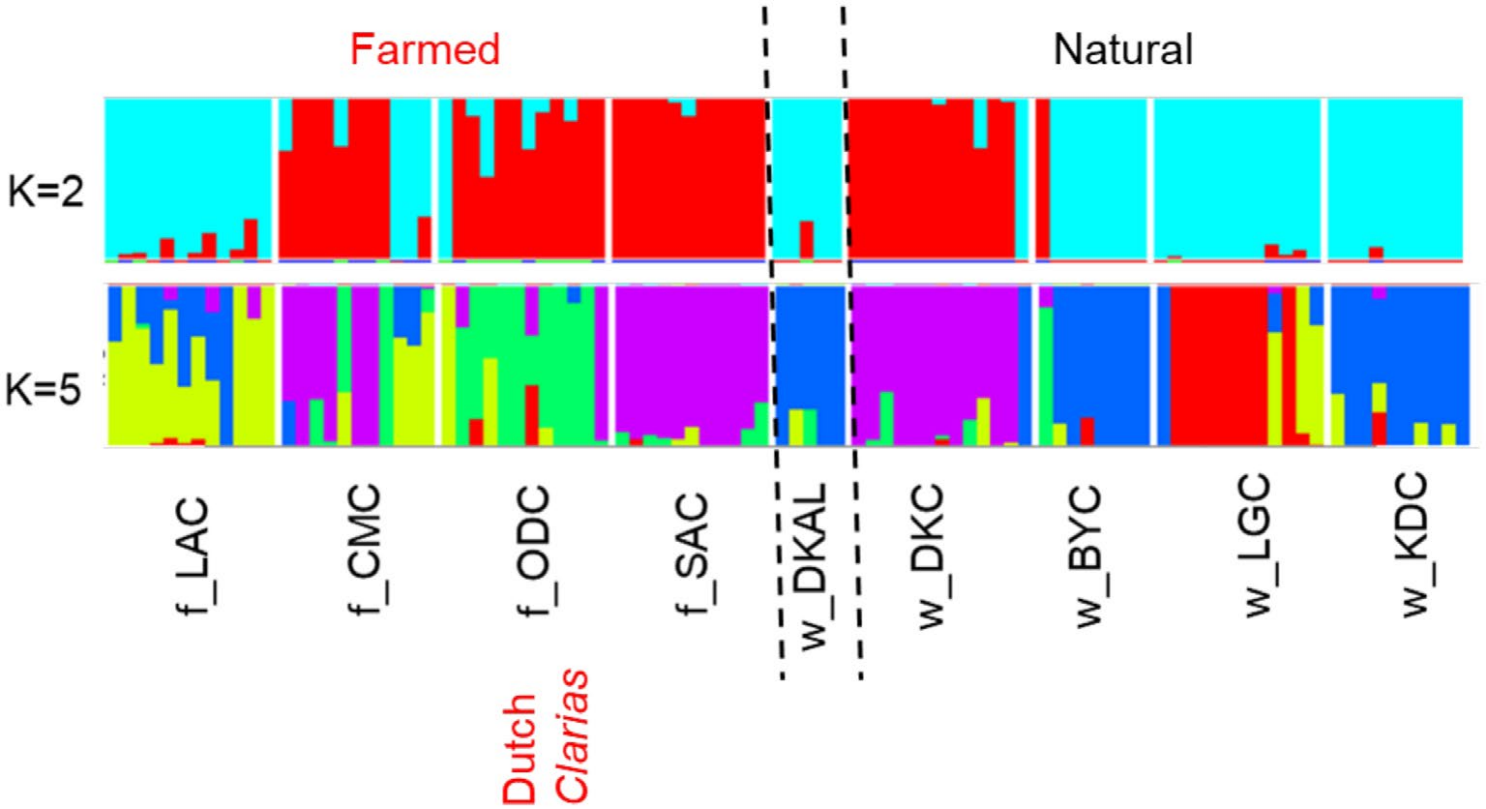
Admixture plot illustrating the genetic ancestry of individual fish samples from farmed and natural *Clarias gariepinus* populations based on SNP data. Each vertical bar in the plot represents an individual fish sample, partitioned into segments corresponding to inferred genetic clusters. The plot is grouped according to farmed (the first four), albino and natural (the last four). At *K* = 2, we tested the hypothesis whether samples can be differentiated into farmed and natural sampling sites. *K* = 5 shows further genetic differentiation among sampling sites and hybridisation across the farmed and natural sampling sites.

For the admixture analysis, at *K* = 2 admixture was more evident among individuals in the farmed sites compared to the natural sites, which had few admixed individuals (Figure 5). There was no clear differentiation between the farmed and natural sampling sites, but the albino natural samples (w_DKAL) shared a similar ancestry component with certain natural sampling sites (w_BYC, w_KDC and w_LGC) and f_LAC from the farmed site. The lowest cross‐validation error value suggested that *K* = 5 best described the population structure. At this value, most of the farmed sites were highly admixed from multiple sources except in the f_SAC with few admixed individuals. The farmed f_SAC from northeast Nigeria was differentiated from their farmed counterparts from the southwest but shared a similar ancestry component with the natural w_DKC and few individuals from the farmed f_CMC. The Dutch *Clarias* (f_ODC) was genetically distinct from all the farmed sampling sites except for some individuals in the f_CMC with which they shared a similar ancestral pattern. The farmed f_LAC showed evidence of admixture from both farmed and natural sampling sites from w_BYC, w_KDC and w_DKAL. For the natural samples, w_BYC, w_KDC and the albino w_DKAL clustered together, and they appeared to be differentiated from w_LGC. The albino natural samples (w_DKAL) clearly clustered with w_BYC and w_KDC, with little evidence of admixture at either of these sites.

### 3.7 Outlier Analysis

**FIGURE 6:**
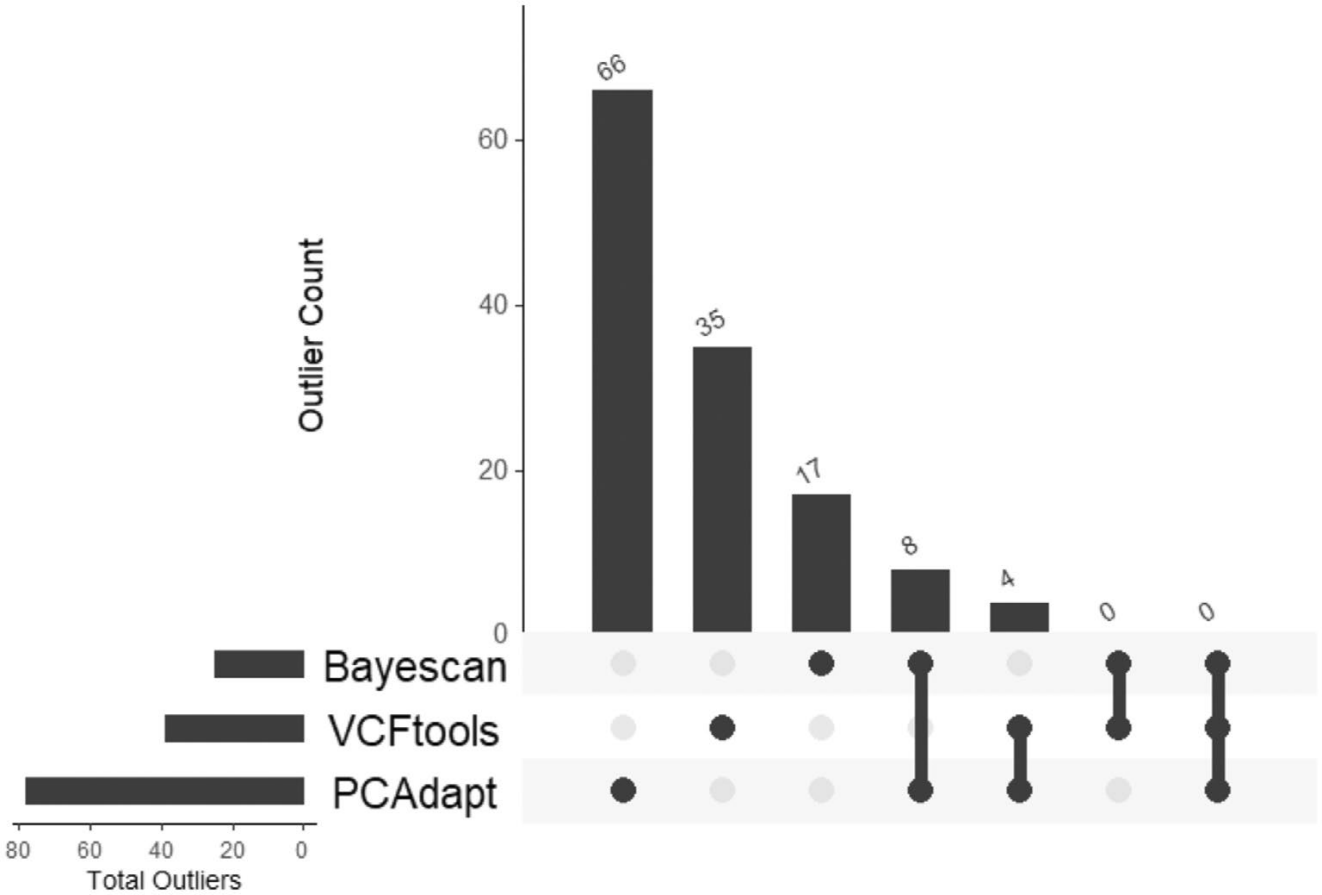
Outlier SNPs from *Bayescan*, VCFtools, and *PCAdapt* analysis. The bar chart shows the outlier count and overlapping outliers are shown under the bars.

*PCAdapt* detected 78 outliers, VCFtools 39 and *Bayescan* 25 (Figure 6). These outliers were spread across multiple chromosomes in the genome. Out of the outliers detected, eight were common between the *Bayescan* and *PCAdapt* analyses, and four between VCFtools and *PCAdapt*. Some of the genes identified were involved in metabolic pathways, response to stimulus, multicellular organismal process, developmental process and negative regulation of multicellular organismal process (Table S6). Gene Ontology (GO) enrichment analysis of outlier loci also revealed significant overrepresentation of terms associated with lipid oxidation and fatty acid β‐oxidation including lipid oxidation (GO:0034440), fatty acid beta‐oxidation using acyl‐CoA dehydrogenase (GO:0033539) and acyl‐CoA dehydrogenase activity (GO:0003995). Additionally, we detected enrichment for cell death pathways such as apoptotic process (GO:0006915) and necroptotic process (GO:0070266). Several terms associated with signal adenylate cyclase‐activating G protein‐coupled receptor signalling pathway (GO:0007189), cAMP biosynthetic process (GO:0006171) and adrenergic receptor binding (GO:0031690). Gene Ontology (GO) enrichment analysis was performed on nine outlier loci identified by *Bayescan* as being under divergent selection (i.e., loci with alpha values ≥ 0 and *q*‐value ≤ 0.05) (Figure S4). The analysis revealed significant enrichment for the biological process ‘necroptotic process’ (GO:0070266; *p*_adj_ = 4.95 × 10^−22^) considered the sole form of programmed cell death during development; and cellular component term ‘BRCA2‐MAGE‐D1 complex’ (GO:0033593; *p*_adj_ = 4.99 × 10^−2^) associated with mediating the synergistic activities of two proteins in regulating cell growth and DNA repair.

## 4 Discussion

In this study, the genetic diversity and differentiation of farmed and natural sampling sites of *C. gariepinus* in Nigeria were investigated using the mtDNA COI gene and 3RAD sequencing approaches. Although population sizes varied across sites and so our sample sizes were uneven, we found limited evidence of admixture between farmed and natural populations, aside from an albino group likely of farmed origin but sampled from the wild. Despite some shared mtDNA haplotypes, the lack of significant genetic differentiation between farmed and natural groups in the AMOVA suggests minimal interbreeding. Encouragingly, natural populations displayed relatively high genetic variation, though with signs of inbreeding exceeding those in farmed populations. Similar findings have been reported in Kenya, where natural populations showed greater haplotype diversity and stronger genetic differentiation from farmed stocks (Barasa et al. 2017; Aminisarteshnizi and Moyo 2024). Our admixture analyses suggested that farmed sites included fish from multiple sources, which may involve intentional or unintentional mixing of individuals from genetically distinct backgrounds to improve traits such as growth (Ibiwoye 2018). There was also some evidence of admixture among natural sites, which could suggest that populations have yet to become isolated to a point that would pose severe risks to their viability. Nevertheless, based on both sharing of mtDNA haplotypes and 3RAD‐based admixture analysis, the five albino individuals sampled from w_DKC are strong candidates for farm escapees, possibly from the f‐SAC dam site, suggesting that there are ongoing risks to the genetic integrity of natural populations unless there are measures implemented to regulate escapes. We thus recommend implementing preventive measures to minimise fish escapes and enacting policies that will mandate conducting risk assessments before introducing non‐native species and strains.

### 4.1 Distribution of Mitochondrial Haplotypes in Farmed vs. Natural Catfish

The mtDNA analyses revealed haplotypes that were only found in natural sampling sites, suggesting limited gene flow between farmed and natural sampling sites. Two of the haplotypes (8 and 9) unique to natural populations that had a 100% match in the BOLD database have previously been reported in a survey of freshwater fish diversity in Israel (Tadmor‐Levi et al. 2023). This suggests a broader geographic distribution of these haplotypes in the Middle East and Asia. Haplotype 5, most commonly found in farmed sampling sites (f_LAC and f_CMC) but also one natural site (w_KDC), was previously reported in the natural environment in southeastern Algeria (Behmene et al. 2022) and southeast Nigeria (Nwani et al. 2011). Similarly, predominantly natural haplotype 7, which was also found in farmed f_LAC, has in previous studies been reported to be a natural haplotype in both North central (Iyiola et al. 2018) and Southeastern Nigeria (Nwani et al. 2011). This could suggest that the farmed stock has been sourced from wild populations; in Nigeria farmers often acquire gravid broodstock from fishers during the peak rainy season when natural *Clarias* spp. are about to begin breeding (M.K.S., personal observation). Previous studies have also indicated that farmed haplotypes have been sourced from the natural environment, as reported in Nigeria (Nwani et al. 2011; Iyiola et al. 2018), Algeria (Behmene et al. 2022) and DR Congo (Sonet et al. 2019). Purchasing broodstock from the natural environment might be preferred due to its cost‐effectiveness compared to buying from farms, if breeders view natural‐caught broodstock as more economically viable, rather than having genetic advantages (Ibiwoye 2018). Nevertheless, the higher pairwise mtDNA nucleotide diversity in some of the farmed when compared to natural populations suggests that the broodstock often includes multiple sources.

The closer genetic relationship of the albino fish from w_DKAL to farmed fish from other sites rather than to the normally‐pigmented samples caught at the same natural site is consistent with escape from farms. The two haplotypes found in these albino samples (10 and 11) were not found in earlier barcoding studies of Nigerian natural populations but have been reported in wild catfish from the Lower and Middle Congo Rivers and major river basins in the Lower Guinean province (Sonet et al. 2019), suggesting that these also could represent farmed escapes from broodstock that has been introduced to multiple countries.

### 4.2 mtDNA Genetic Diversity and Differentiation

The mtDNA COI results indicate that natural sites generally contain a higher diversity of haplotypes compared to fish farms, which rely on a limited number of broodstock but suggest that wild populations in the northeast of Nigeria might need additional protection to ensure their viability. However, overall genetic diversity in this study was low, even when compared to previous mtDNA COI studies that were restricted to relatively limited geographic regions such as ours. For example, also based on COI, Kundu et al. (2023) reported 20 haplotypes with 81 segregating sites, haplotype diversity (*H*_d_ = 0.854) and nucleotide diversity (*pi* = 0.258) among 63 samples collected from three sampling sites along the Nyong River in Cameroon. A similar study conducted in three rivers in southwest Nigeria using the mtDNA *cytb* gene (Popoola 2022) revealed 53 haplotypes among 54 individuals sampled, with greater haplotype diversity (*H*_d_ = 0.999) and nucleotide diversity (*pi* = 0.073) than the current study. Nyunja et al. (2017) studied five *C. gariepinus* hatchery populations in Kenya, and found 33 haplotypes among 93 individuals sampled, 60 segregating sites, high haplotype diversity (*H*_d_ = 0.988 ± 0.031), but low nucleotide diversity (*pi* = 0.024 ± 0.026). Alal et al. (2021) suggested that the genetic diversity in small *C. gariepinus* populations in the wild as observed in Lake Kenyatta, Kenya, can be improved through stock augmentation by conservation scientists. For example, this approach could be used to boost declining *C. gariepinus* in lakes due to overfishing and periodic drying. Sharing of mtDNA haplotypes between farmed and natural populations that could suggest sourcing of broodstock from the wild has also been reported in Lakes Victoria, Kanyaboli and Baringo (Barasa et al. 2017). Overall, while it is encouraging that there are still a large number of maternal haplotypes maintained in the natural population, ongoing collection of wild individuals for farming could provide a risk to population viability in the future.

Patterns of genetic differentiation between farmed and wild populations are also similar to those found in other studies. In the AMOVA analysis we found high genetic differentiation among the nine populations (global *F*_st_ = 0.408), with most of the genetic variation explained by differences within populations and among populations within farmed or natural categories, rather than between farmed and natural populations, regardless of whether the albino individuals were included. This is similar to a study involving farmed and natural *C. gariepinus* in Nigeria reported by Suleiman et al. (2020), who also observed that most of the genetic variation was explained within populations rather than between the categories, based on Random Amplified Polymorphic DNA (RAPD) markers. However, focusing only on natural *C. gariepinus* populations in Nigeria, Popoola (2022) found most of the genetic variation among rather than within the three populations sampled. Collectively, these studies have shown that the COI gene, which is primarily used as a barcoding marker, can be applied to successfully differentiate different populations of *C. gariepinus* . This cheap molecular approach compared to deep sequencing could be a vital tool for the conservation of *C. gariepinus* in Nigeria.

### 4.3 3RAD Genetic Diversity in Farmed and Natural Populations

The genome‐wide SNP data revealed higher genetic diversity in natural populations than in farmed ones, aligning with the mtDNA COI results. This reduced diversity in farms likely reflects selective breeding and limited broodstock, as observed in other farmed fish (Zeinab et al. 2014). Preserving genetic diversity requires genetically diverse broodstock (Fernández et al. 2014), but the Nigerian catfish industry faces challenges in accessing quality broodstock (Adeleke et al. 2021). Effective broodstock management through controlled reproduction is essential (Duncan et al. 2013), yet widespread indiscriminate breeding undermines this goal (Sanda et al. 2024).

Overall diversity based on the 3RAD data were also low, suggesting limited variation within populations. This may result from the observed small effective population sizes for most of the populations, founder effects, or selective pressures in both natural and farmed environments. All the farmed and natural sites were estimated to have a low effective population size (less than the sample size), except for f_LAC (*N*_e_ = 83.2). Infinite *N*_e_ observed in f_ODC suggests that within the resolution of our dataset, the estimated *N*_e_ is beyond the upper limit in the linkage disequilibrium method to detect any signal of genetic drift of breeding individuals (Do et al. 2014). Meanwhile, the high *N*_e_ estimate observed in w_LGC (*N*_e_ = 9497.7) contrasts sharply with smaller estimates from both farmed and natural sites at other locations. Lake Geriyo (w_LGC) is known for year‐round fishing and high catches during the rainy season (Kefas et al. 2025), which likely supports a large census population size contributing to a higher *N*_e_. Sampling from this site was easy especially when compared to the other natural sites. Low effective population size threatens the long‐term sustainability of species. For example, RAD sequencing data from farmed turbot, gilthead seabream, European seabass and common carp has shown that small effective population sizes (≤ 50 fish) in a breeding population may put the sustainability of the farming programs at risk (Saura et al. 2021). Likewise, overexploited and fragmented natural populations may lose adaptive potential, as witnessed in New Zealand snapper ( *Pagrus auratus* ), where genetic diversity declined and effective population sizes were drastically lower than census population sizes (Hauser et al. 2002). Populations with lower *N*_e_ are highly susceptible to genetic drift and loss of genetic diversity including inbreeding depression, deleterious mutations and reduced adaptive capacity (Mamoozadeh et al. 2025).

Overexploitation, environmental change, pollution and habitat destruction are among the main causes of population decline that are increasingly threatening fish diversity in the natural environment (Martinez et al. 2018; Petit‐Marty et al. 2022). These threats were noticeable in some of the natural sampling sites in this study, such as the River Benue Yola, which experiences seasonal drying during prolonged dry periods. The review by Emmanuel et al. (2021) highlighted the impacts of overexploitation, habitat fragmentation and pollution on fish diversity in Nigeria. Strengthening fisheries management, regulation of fishing activities and implementing habitat restoration are therefore important measures for maintaining the genetic diversity of natural *C. gariepinus* in Nigeria.

The high *F*_is_ in the natural sampling sites in the present study could also be due to declining population sizes leading to an increase in the likelihood of mating between related individuals. This was reported in the giant fish *Arapaima gigas* in the Amazon, which experienced a loss of genetic diversity and high inbreeding rates caused by illegal fishing and environmental pressure reducing the effective population size (Fazzi‐Gomes et al. 2021). To mitigate inbreeding in natural *C. gariepinus* populations, sustainable fishing practices and conservation efforts that prioritise genetic diversity and enhance gene flow through habitat connectivity are essential. Inbreeding in natural *C. gariepinus* could be reduced by embracing sustainable fishing practices and conservation efforts that prioritise genetic diversity and promote gene flow through habitat connectivity.

### 4.4 Genetic Differentiation Among Farmed and Natural Populations

The consistently high genetic differentiation found using the SNP data between farmed and natural *C. gariepinus* suggests limited mixing between the two groups. However, as for the mtDNA results, the albino individuals (w_DKAL), though sampled from the wild, showed genetic similarity to farmed populations, again suggesting they had escaped from farm stocks. This pattern aligns with results from previous studies where farmed escapes showed higher similarity to their source populations based on microsatellite markers (Erkinaro et al. 2010; Šegvić‐Bubić et al. 2016). Based on predictive modelling of a 14 year dataset of farmed escapee abundance from 54 rivers, Mahlum et al. (2021) further revealed that the frequency of morphologically identified escapees in Atlantic salmon *Salmo salar* in western Norwegian rivers was linked to the intensity of local aquaculture. If the albino sample is considered as farmed, it can be concluded based on the PCA that *C. gariepinus* populations in Nigeria, despite their low genetic diversity, can be differentiated using the genome‐wide SNPs into fish of farmed and natural origin. The significant *F*_st_ values between farmed and natural sampling sites are an important indicator for the implementation of conservation strategies that will manage these different farmed and natural sites separately to preserve their unique diversity. Such data are relevant for guiding effective fisheries and aquaculture management (Kemp et al. 2023).

Admixture analyses provided evidence of some mixing between sites for both farmed and natural populations, which could reflect ongoing gene flow or transport of samples between sites. Among the natural sites, Lake Geriyo and Dadin Kowa Dam are both linked to the River Benue system, facilitating fish movement and gene flow (Essien et al. 2019; Hassan et al. 2015; Meulenbroek et al. 2024). The transport of farmed fish, including broodstock and fingerlings from different sources, is a common practice among farmers in Nigeria (Ibiwoye 2018). Although some broodstock are collected from private fish farms, it is not uncommon to source them from natural water bodies depending on cost, availability and desired traits (Eze et al. 2022; Mekuleyi et al. 2022; Eyo et al. 2021). Overall, the *F*_st_ values suggest that even after years of *C. gariepinus* farming in Nigeria, there is still a noticeable degree of genetic differentiation maintained across different farm and natural sites.

### 4.5 Outlier Loci and Enrichment Analysis

Identification of outlier loci from multiple genome scan approaches Gene Ontology (GO) enrichment analysis revealed a significant overrepresentation of genes involved in lipid metabolism, cell signalling and apoptotic processes. These results suggest that pathways related to metabolic regulation and cellular communication pathways may be key targets of selection in response to environmental pressures. Metabolites expand their signalling influence to processes outside metabolism including nutrient sensing and storage, embryonic development, cell survival and differentiation and immune activation and cytokine secretion (Baker and Rutter 2023). Habitat degradation in Nigerian freshwater systems may drive such adaptations by limiting nutrient availability and triggering metabolic resilience strategies (Baker and Rutter 2023). At the systemic level this can often be interpreted as an attempt to delay or cease growth and reproduction in order to persevere for future periods of increased resource abundance (Kapahi 2010; Austad and Hoffman 2018). Outlier loci functionally enriched for genes related to lipid oxidation and programmed cell death were detected, suggesting that selection may be acting on metabolic and immune‐related pathways, potentially contributing to variation between farmed and natural *C. gariepinus* or fitness differences across sampling sites. Loci associated with signal adenylate cyclase‐activating G protein‐coupled receptor signalling pathway, cAMP biosynthetic process and adrenergic receptor binding suggest that outlier loci are functionally enriched in pathways involved in metabolic regulation, immune signalling and cellular homeostasis, which may reflect different selective pressures in the farmed and wild sampling sites. G‐protein‐coupled receptors are proteins sharing a seven α‐helical transmembrane structure that controls several physiological processes in both vertebrates and invertebrates and have been linked to insecticide resistance (Liu et al. 2021). The outlier loci under divergent selection were enriched in the necroptotic process BRCA2‐MAGE‐D1 complex, suggesting that divergent selection may be acting on genes involved in cell death regulation and transcriptional regulation, potentially reflecting adaptive responses to environmental or physiological stressors (Vandenabeele et al. 2010; Tian et al. 2005).

## 5 Conclusions

We have provided an assessment of genetic diversity, population structure and differentiation between farmed and natural *C. gariepinus* populations in Nigeria. These genetic insights serve as indicators of population health including levels of inbreeding and adaptive capacity to environmental change. Despite some variation, overall genetic diversity was low across both farmed and natural populations. Natural populations generally retained their genetic integrity, with limited admixture, whereas farmed populations showed evidence of extensive mixing from multiple sources. The combination of small effective population size, low genetic diversity, evidence of admixture and farm escapees highlights the urgent need for better management and conservation strategies. In aquaculture, maintaining genetic diversity requires better broodstock management including expanding breeding populations and avoiding repeated use of a few broodstock. These findings emphasise the importance of integrating genetic monitoring into fisheries and aquaculture management. Future research directions should consider sampling across more geographical locations and farms to further understand the dynamics of genetic diversity and population structure in farmed and natural fish.

## Funding

The research reported here was funded by a PhD scholarship to M.K.S. from the Commonwealth Scholarship Commission and the Foreign, Commonwealth and Development Office in the UK. All views expressed here are those of the authors, not the funding body.

## Conflicts of Interest

The authors declare no conflicts of interest.

## Supporting information


**Appendix S1:** eva70204‐sup‐0001‐AppendixS1.docx.

## Data Availability

Raw sequence data were deposited in the European Nucleotide Archive (ENA) SRA (PRJEB78629). Mitochondrial DNA cytochrome c oxidase 1 (CO1) sequences have been deposited to GenBank, with accession numbers PQ197861–PQ197964. All additional data including scripts and metadata are available at https://github.com/mksanda/Clarias_gariepinus_3RAD.
