# Supplementary material for: Genetic Diversity, Population Structure and Differentiation of Farmed and Wild African Catfish (Clarias gariepinus) in Nigeria
Source: Evol Appl. 2026 Jan 30;19(2):e70204. doi: 10.1111/eva.70204 (PMC12858669; doi:10.1111/eva.70204)
Supplement: Supplementary file 1 — Appendix S1: eva70204‐sup‐0001‐AppendixS1.docx. [file EVA-19-e70204-s001.docx]

**
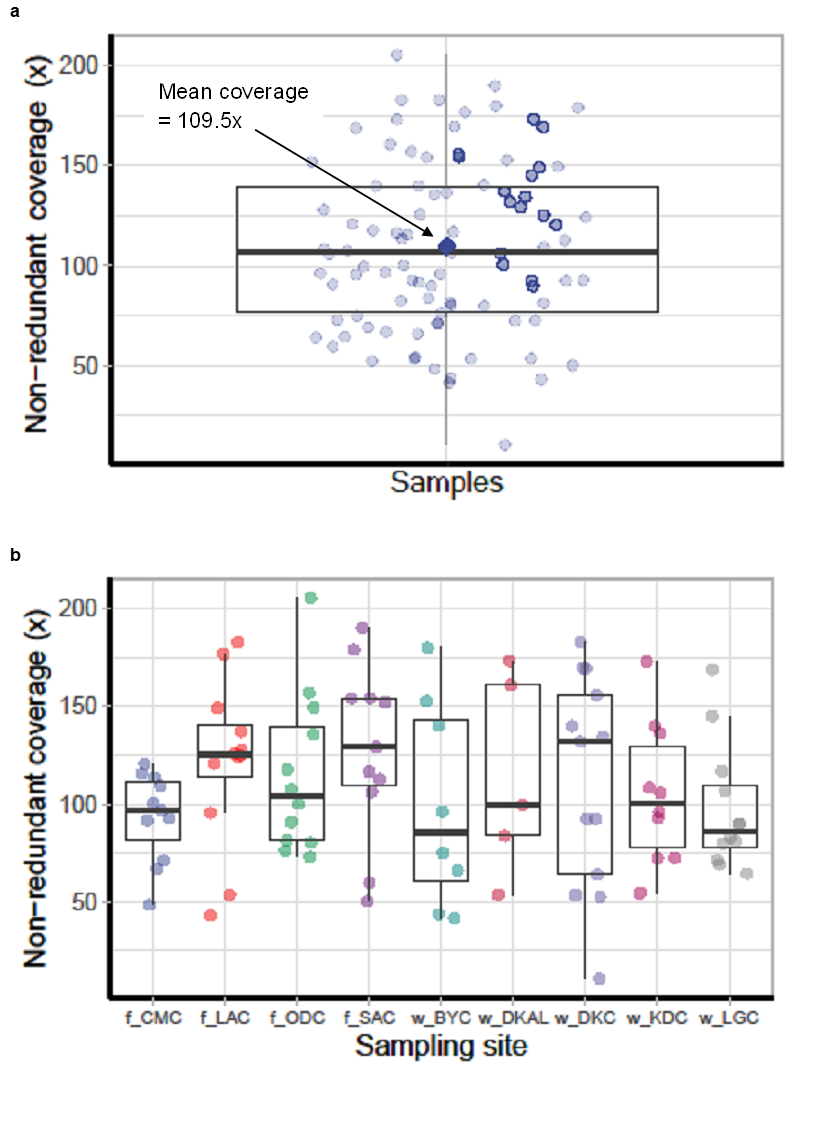
**

**Supplementary Figure 1.** Boxplot illustrating the distribution of non-redundant read coverage indicating the number of reads mapped to specific genomic regions or features for each population for individual samples with mean coverage (a), and distribution of reads across sampling sites (b). The boxplot provides a visual summary of the read distribution, with the box indicating the interquartile range (IQR) and the median read coverage, and the whiskers extending to the minimum and maximum values or to a specified range. The prefixes f and w, on the x-axis indicate farmed and wild samples, respectively.

**
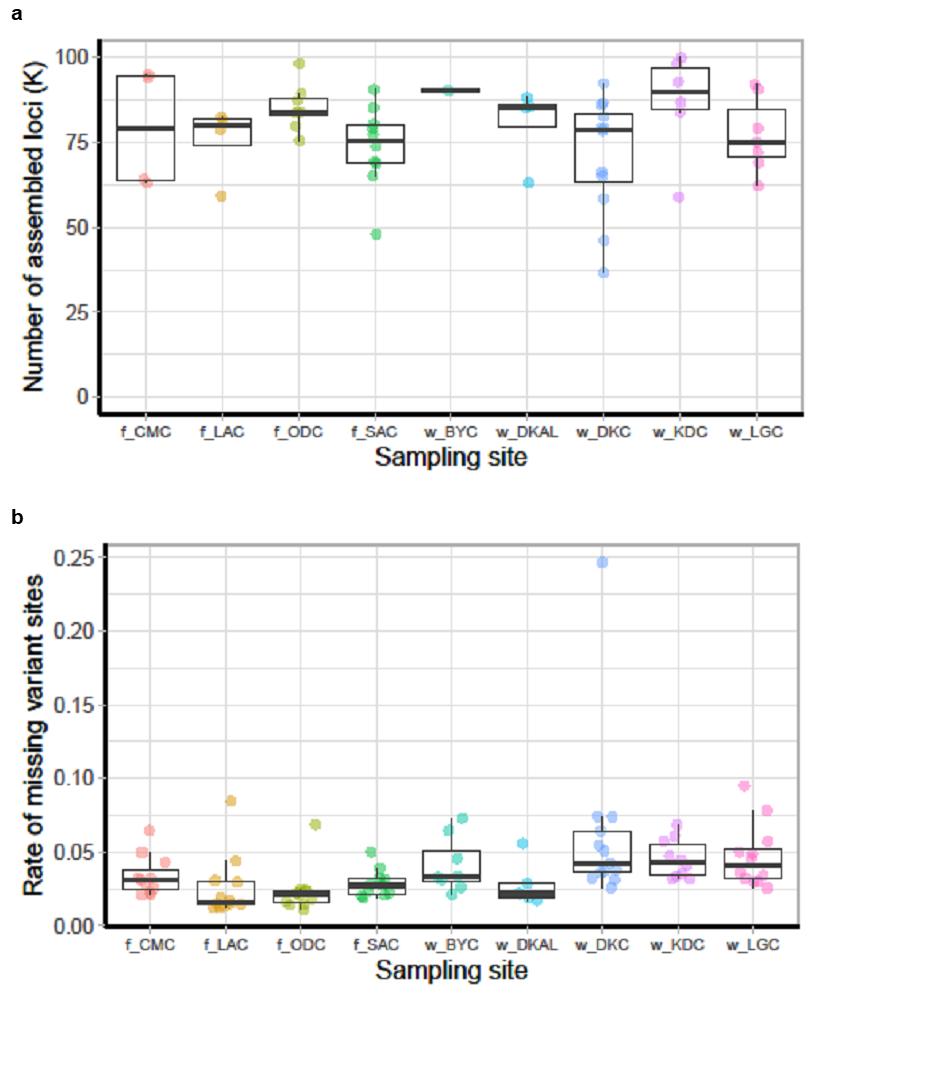
**

**Supplementary Figure 2.** Boxplot illustrating the distribution of assembled loci across different sampling locations (a) and the rate of missing variant sites (b) for the 3RAD sequencing data. The y-axis indicates the number of loci in thousands and the x-axis represents the sampling locations. Each point on the plot represents a sample within a location. The prefixes f and w, on the x-axis indicate farmed and wild samples, respectively.


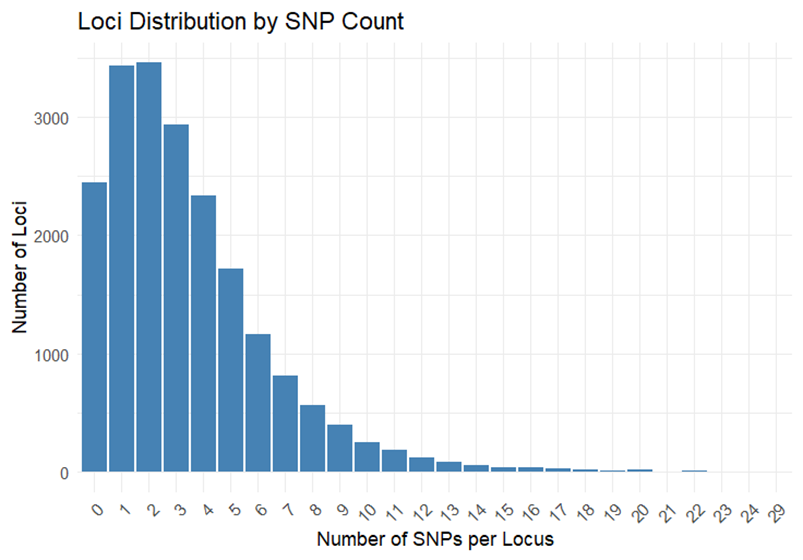


**Supplementary Figure 3.** Distribution of the number of SNPs per catalog locus


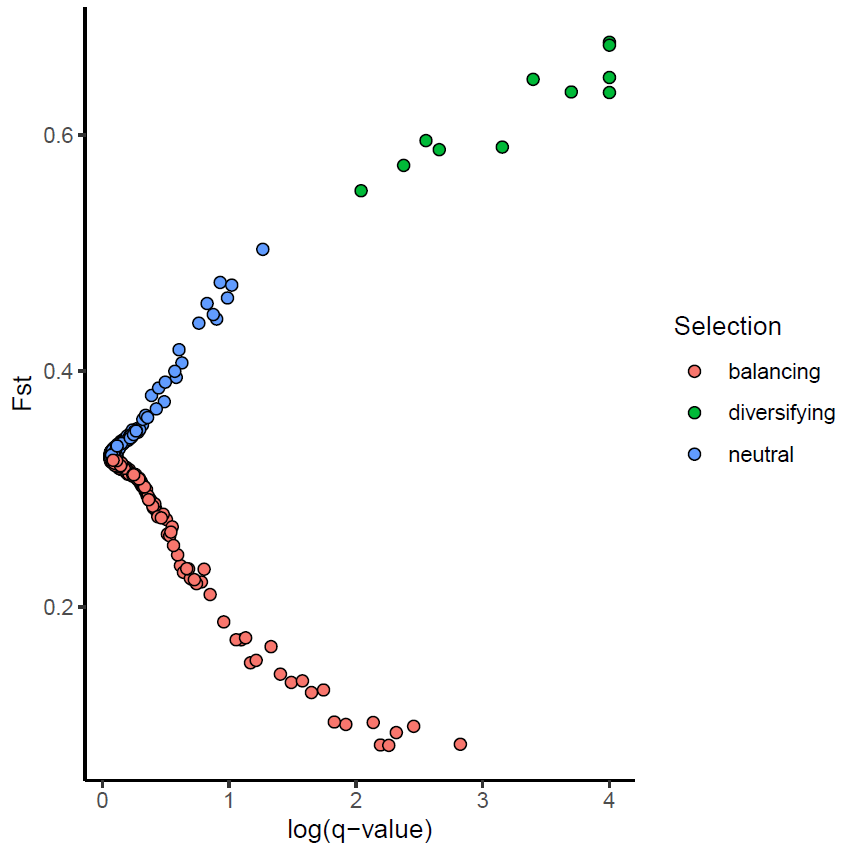


**Supplementary Figure 4.** Bayescan outlier analysis showing loci under putative neutral, balancing, and diversifying selection

**Supplementary Table 1. Global distribution of haplotypes based on 100% match from BOLD Systems search.**

| **Haplotype** | **Country and reference** |
| --- | --- |
| 5 | Nigeria (n = 2): MG824580, MG824583 (Iyiola et al., 2018), BAFEN141-10 (Nwani et al., 2011); Algeria (n = 4): ON643478, ON643477, ON643476, ON643475 (Behmene et al., 2022); Egypt (n = 1): MK335911 (unpublished) |
| 7 | Nigeria (n = 2): MG824581 (Iyiola et al., 2018), BAFEN140-10 (Nwani et al., 2011) |
| 8 | Israel (n = 11): FWISR057-21, FWISR056-21, FWISR055-21, FWISR054-21, FWISR053-21, FWISR052-21, FWISR046-21, LKCOX059-19, LKCOX058-19, LKCOX057-19, LKCOX056-19 (Tadmor-Levi et al., 2023); Thailand (n = 3): JF292311, JF292314, MT571809 (Wong et al., 2011); Bangladesh (n = 1): MG988400 (unpublished); Egypt (n = 2): MK335909, MK335910, (Unpublished); Nigeria (n = 12): BAFEN129-10, BAFEN130-10, BAFEN131-10, BAFEN134-10, BAFEN137-10, BAFEN144-10, BAFEN145-10, BAFEN146-10, BAFEN147-10, BAFEN148-10, BAFEN150-10, BAFEN151-10 (Nwani et al., 2011); Syria (n = 1): FFMBH2002-14 (Geiger et al., 2014); India (n = 1): FNWG200-16 (Patil et al., 2018) |
| 9 | Israe (n = 7): FWISR057-21, FWISR056-21, FWISR055-21, FWISR054-21, FWISR053-21, FWISR052-21, FWISR046-21 (Tadmor-Levi et al., 2023); Thailand (n = 1): JF292314 (Wong et al., 2011); Bangladesh (n = 1): MG988400 (unpublished); Egypt (n = 1): MK335909, MK335910 (Unpublished) |
| 10 | DR Congo (n = 5): BCOVR501-17 (Sonet et al., 2019), DCF305-15, DCF751-15, DCF752-1, DCF602-15 (unpublished); Brazil (n = 6): FUPR532-09, LBPV-31863, LBPV-31864, LBPV-31865, LBPV-31866, FUPR536-09 (Pereira et al., 2013); |
| 11 | DR Congo (n = 1) BIN ID: AAB2256 (Sonet et al., 2019) |

**Supplementary 2. Comparison of mtDNA summary statistics (see Table 3) between farmed and natural *Clarias gariepinus* for three alternative scenarios: when the w_DKAL albino population considered as natural, excluded from the analyses, or treated as farmed. The columns show statistical information including t-statistics and associated p-values, degrees of freedom (d.f.), and the 95% lower (CI lower) and upper bound (CI upper) confidence intervals.**

| **Parameter** | **Farmed** | **Natural** | **t-statistic** | **p-value** | **d.f.** | **95% CI lower** | **95% CI upper** |
| --- | --- | --- | --- | --- | --- | --- | --- |
| Na (albino as natural) | 2.5 | 3.6 | -1.540 | 0.168 | 6.893 | -2.794 | 0.594 |
| Na (without albino) | 2.5 | 4.0 | -2.324 | 0.061 | 5.769 | -3.095 | 0.094 |
| Na (albino as farmed) | 2.4 | 4.0 | -2.799 | 0.027 | 6.815 | -2.959 | -0.241 |
| S (albino as natural) | 12.5 | 4.8 | 1.776 | 0.169 | 3.178 | -5.668 | 21.068 |
| S (without albino) | 12.5 | 4.5 | 1.835 | 0.157 | 3.246 | -5.295 | 21.295 |
| S (albino as farmed) | 11.2 | 4.5 | 1.831 | 0.134 | 4.468 | -3.054 | 16.454 |
| Hd (albino as natural) | 0.321 | 0.647 | -2.496 | 0.078 | 3.416 | -0.715 | 0.063 |
| Hd (without albino) | 0.321 | 0.659 | -2.548 | 0.070 | 3.598 | -0.723 | 0.047 |
| Hd (albino as farmed) | 0.376 | 0.659 | -2.357 | 0.065 | 4.971 | -0.590 | 0.026 |
| pi (albino as natural) | 0.005 | 0.003 | 0.861 | 0.443 | 3.581 | -0.005 | 0.010 |
| pi (without albino) | 0.005 | 0.002 | 1.196 | 0.316 | 3.060 | -0.005 | 0.011 |
| pi (albino as farmed) | 0.005 | 0.002 | 1.611 | 0.180 | 4.133 | -0.002 | 0.009 |

**Supplementary Table 3. Analysis of Molecular Variance (AMOVA) of mtDNA COI sequences in farmed and natural *C. gariepinus* populations from northeastern and southwestern Nigeria including albino as natural samples**

| **Source of Variation** | **d.f.** | **SS** | **Var** | **% Var** |
| --- | --- | --- | --- | --- |
| Among groups | 2 | 8.992 | -0.046 Va | -11.17 |
| Among populations within groups | 8 | 18.083 | 0.169 Vb | 41.35 |
| Within populations | 200 | 57.118 | 0.286 Vc | 69.82 |
| Total | 210 | 84.123 | 0.409 | 100 |

Notes: SS, sum of squares; Var, variance component; Va, variance components among populations; Vb, variance components among populations within groups; Vc, variance components within populations

**Supplementary Table 4. Analysis of Molecular Variance (AMOVA) of mtDNA COI sequences in farmed and natural *C. gariepinus* populations from northeastern and southwestern Nigeria without including the albino sample**

| **Source of Variation** | **d.f.** | **SS** | **Var** | **% Var** |
| --- | --- | --- | --- | --- |
| Among groups | 2 | 9.995 | -0.039 Va | -9.35 |
| Among populations within groups | 7 | 17.278 | 0.174 Vb | 42.16 |
| Within populations | 192 | 53.302 | 0.278 Vc | 67.19 |
| Total | 201 | 80.575 | 0.413 | 100 |

Notes: SS, sum of squares; Var, variance component; Va, variance components among populations; Vb, variance. components among populations within groups; Vc, variance components within populations.

**Supplementary Table 5. Analysis of Molecular Variance (AMOVA) of mtDNA COI sequences in nine farmed and natural *C. gariepinus* populations from northeastern and southwestern Nigeria including albino as farmed samples**

| **Source of Variation** | **d.f.** | **SS** | **Var** | **% Var** |
| --- | --- | --- | --- | --- |
| Among groups | 2 | 10.338 | -0.036 Va | -8.59 |
| Among populations within groups | 8 | 18.083 | 0.170 Vb | 41.12 |
| Within populations | 200 | 55.702 | 0.279 Vc | 67.47 |
| Total | 210 | 84.123 | 0.413 | 100 |

Notes: SS, sum of squares; Var, variance component; Va, variance components among populations; Vb, variance components among populations within groups; Vc, variance components within populations

**Supplementary Table 6. Significantly enriched Gene Ontology (GO) terms associated with loci identified as outliers. Molecular function (MF) showing biological process (BP), and cellular component (CC), with adjusted p-values (Padj) indicating statistical significance**

| **Source** | **Term ID** | **Term Name** | **Padj** |
| --- | --- | --- | --- |
| GO:MF | GO:0003824 | catalytic activity | 8.200x10-6 |
| GO:MF | GO:1901363 | heterocyclic compound binding | 1.622x10-5 |
| GO:MF | GO:0042802 | identical protein binding | 7.337x10-4 |
| GO:MF | GO:0015662 | P-type ion transporter activity | 3.228x10-3 |
| GO:MF | GO:0003995 | acyl-CoA dehydrogenase activity | 1.302x10-2 |
| GO:MF | GO:0004030 | aldehyde dehydrogenase [NAD(P)+] activity | 3.958x10-2 |
| GO:MF | GO:0051538 | 3 iron, 4 sulfur cluster binding | 4.007x10-2 |
| GO:MF | GO:0003994 | aconitate hydratase activity | 4.007x10-2 |
| GO:MF | GO:0031690 | adrenergic receptor binding | 4.734x10-2 |
| GO:BF | GO:0050896 | response to stimulus | 2.602x10-7 |
| GO:BP | GO:0032501 | multicellular organismal process | 2.775x10-7 |
| GO:BP | GO:0044281 | small molecule metabolic process | 2.817x10-5 |
| GO:BP | GO:0051241 | negative regulation of multicellular organismal process | 1.803×10^-2^ |
| GO:BF | GO:0065008 | regulation of biological quality | 6.715x10-5 |
| GO:BP | GO:0070266 | necroptotic process | 6.747x10-5 |
| GO:BP | GO:0006171 | cAMP biosynthetic process | 1.930x10-4 |
| GO:BP | GO:0032502 | developmental process | 1.731x10-3 |
| GO:BP | GO:0006915 | apoptotic process | 1.791x10-3 |
| GO:BP | GO:0006958 | complement activation, classical pathway | 2.651x10-3 |
| GO:BP | GO:0019220 | regulation of phosphate metabolic process | 2.653x10-3 |
| GO:BP | GO:0007417 | central nervous system development | 7.839x10-3 |
| GO:BP | GO:0006629 | lipid metabolic process | 2.344x10-2 |
| GO:BP | GO:0034440 | lipid oxidation | 3.080x10-2 |
| GO:CC | GO:0031982 | vesicle | 2.692x10-6 |
| GO:CC | GO:0071944 | cell periphery | 2.777x10-4 |
| GO:CC | GO:0045202 | synapse | 3.332x10-3 |
| GO:CC | GO:0042995 | cell projection | 4.434x10-3 |
| GO:CC | GO:0042627 | chylomicron | 5.793x10-3 |
| GO:CC | GO:0005739 | mitochondrion | 6.887x10-3 |
| GO:CC | GO:0034361 | very-low-density lipoprotein particle | 2.628x10-2 |

**References**

Behmene, I. E., Bachir Bouiadjra, B., Homrani, A., Daoudi, M., Sánchez-Vázquez, F. J., López-Lopez, A., Asensio-Pérez, A. I. & Galián, J. 2022. Morphometric and genetic diversity of an African catfish (*Clarias gariepinus*) population from Southeast Algeria. *African Journal of Ecology,* 60**,** 1287-1292. <https://doi.org/10.1111/aje.13055>

Geiger, M. F., Herder, F., Monaghan, M. T., Almada, V., Barbieri, R., Bariche, M., Berrebi, P., Bohlen, J., Casal-Lopez, M., Delmastro, G. B., Denys, G. P. J., Dettai, A., Doadrio, I., Kalogianni, E., Kärst, H., Kottelat, M., Kovačić, M., Laporte, M., Lorenzoni, M., Marčić, Z., Özuluğ, M., Perdices, A., Perea, S., Persat, H., Porcelotti, S., Puzzi, C., Robalo, J., Šanda, R., Schneider, M., Šlechtová, V., Stoumboudi, M., Walter, S. & Freyhof, J. 2014. Spatial heterogeneity in the Mediterranean Biodiversity Hotspot affects barcoding accuracy of its freshwater fishes. *Molecular Ecology Resources,* 14**,** 1210-1221. <https://doi.org/10.1111/1755-0998.12257>

Iyiola, O. A., Nneji, L. M., Mustapha, M. K., Nzeh, C. G., Oladipo, S. O., Nneji, I. C., Okeyoyin, A. O., Nwani, C. D., Ugwumba, O. A. & Ugwumba, A. A. 2018. DNA barcoding of economically important freshwater fish species from north‐central Nigeria uncovers cryptic diversity. *Ecology and Evolution,* 8**,** 6932-6951. <https://doi.org/10.1002/ece3.4210>

Nwani, C. D., Becker, S., Braid, H. E., Ude, E. F., Okogwu, O. I. & Hanner, R. 2011. DNA barcoding discriminates freshwater fishes from southeastern Nigeria and provides river system-level phylogeographic resolution within some species. *Mitochondrial DNA,* 22 Suppl 1**,** 43-51. <https://doi.org/10.3109/19401736.2010.536537>

Patil, T. S., Jamdade, R. A., Patil, S. M., Govindwar, S. P. & Muley, D. V. 2018. DNA barcode based delineation of freshwater fishes from northern Western Ghats of India, one of the world’s biodiversity hotspots. *Biodiversity and Conservation,* 27**,** 3349-3371. 10.1007/s10531-018-1604-0

Pereira, L. H. G., Hanner, R., Foresti, F. & Oliveira, C. 2013. Can DNA barcoding accurately discriminate megadiverse Neotropical freshwater fish fauna? *BMC Genetics,* 14**,** 20. 10.1186/1471-2156-14-20

Sonet, G., Snoeks, J., Nagy, Z. T., Vreven, E., Boden, G., Breman, F. C., Decru, E., Hanssens, M., Ibala Zamba, A., Jordaens, K., Mamonekene, V., Musschoot, T., Van Houdt, J., Van Steenberge, M., Lunkayilakio Wamuini, S. & Verheyen, E. 2019. DNA barcoding fishes from the Congo and the Lower Guinean provinces: Assembling a reference library for poorly inventoried fauna. *Molecular Ecology Resources,* 19**,** 728-743. <https://doi.org/10.1111/1755-0998.12983>

Tadmor-Levi, R., Feldstein-Farkash, T., Milstein, D., Golani, D., Leader, N., Goren, M. & David, L. 2023. Revisiting the species list of freshwater fish in Israel based on DNA barcoding. *Ecology and Evolution,* 13**,** e10812. <https://doi.org/10.1002/ece3.10812>

Wong, L. L., Peatman, E., Lu, J., Kucuktas, H., He, S., Zhou, C., Na-nakorn, U. & Liu, Z. 2011. DNA barcoding of catfish: Species authentication and phylogenetic assessment. *PLOS ONE,* 6**,** e17812. 10.1371/journal.pone.0017812
